# Supplementary material for: Transglutaminase 2 associated with PI3K and PTEN in a membrane-bound signalosome platform blunts cell death
Source: Cell Death Dis. 2023 Mar 28;14(3):217. doi: 10.1038/s41419-023-05748-6 (PMC10050012; doi:10.1038/s41419-023-05748-6)

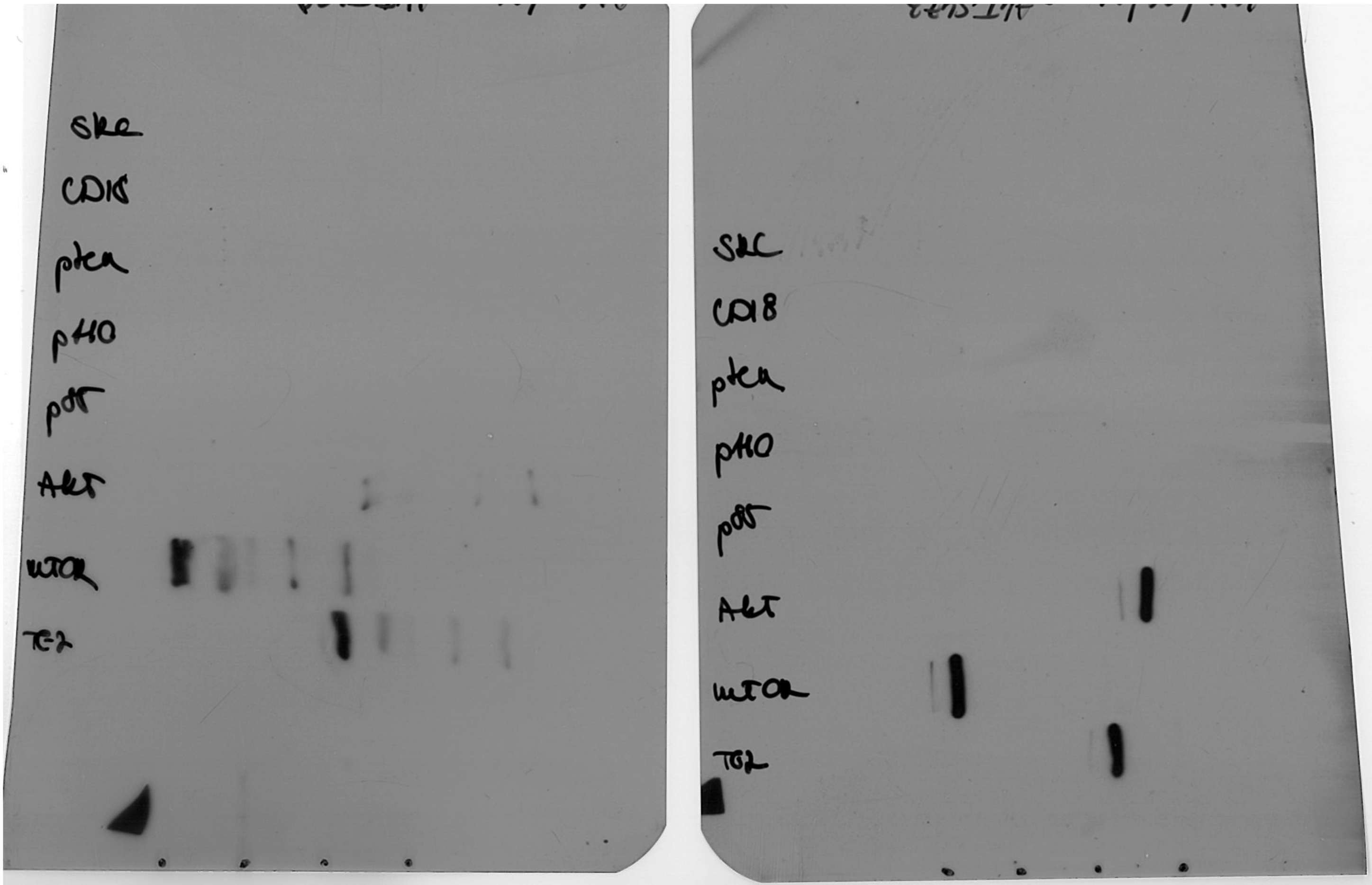
SRC
CD18
pten
p110
Akt
mTOR
TC-2
SRC
CD18
pten
p110
Akt
mTOR
TC2

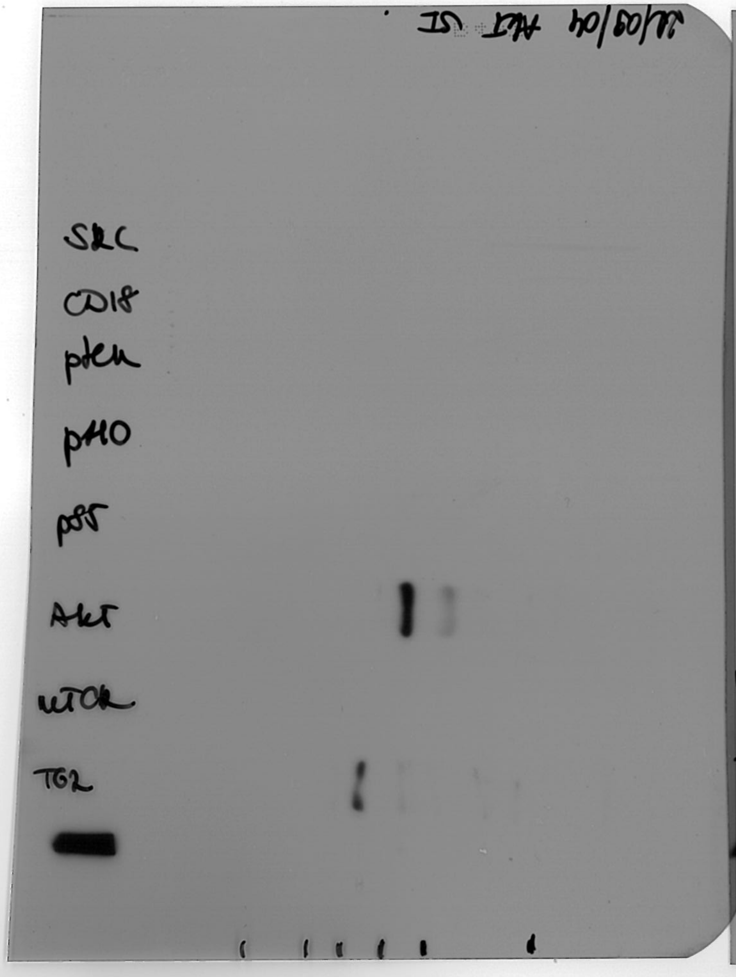
AKT SI
SRC
CD18
pten
p110
p85
Akt
mTOR
TG2

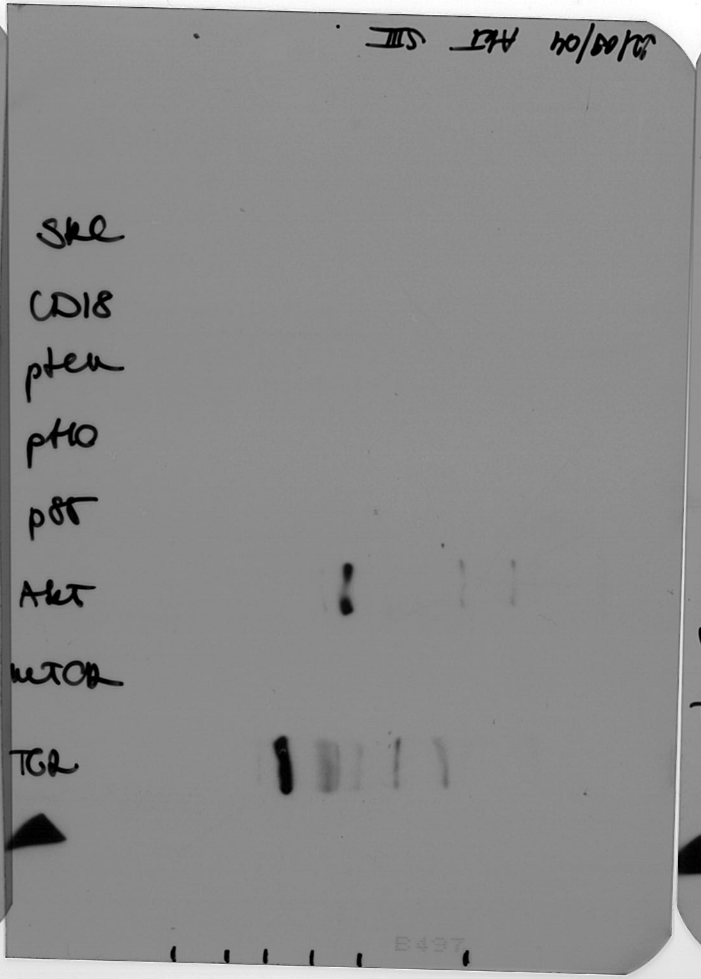
AKT SIII
SRC
CD18
pten
p110
p85
Akt
mTOR
TG2

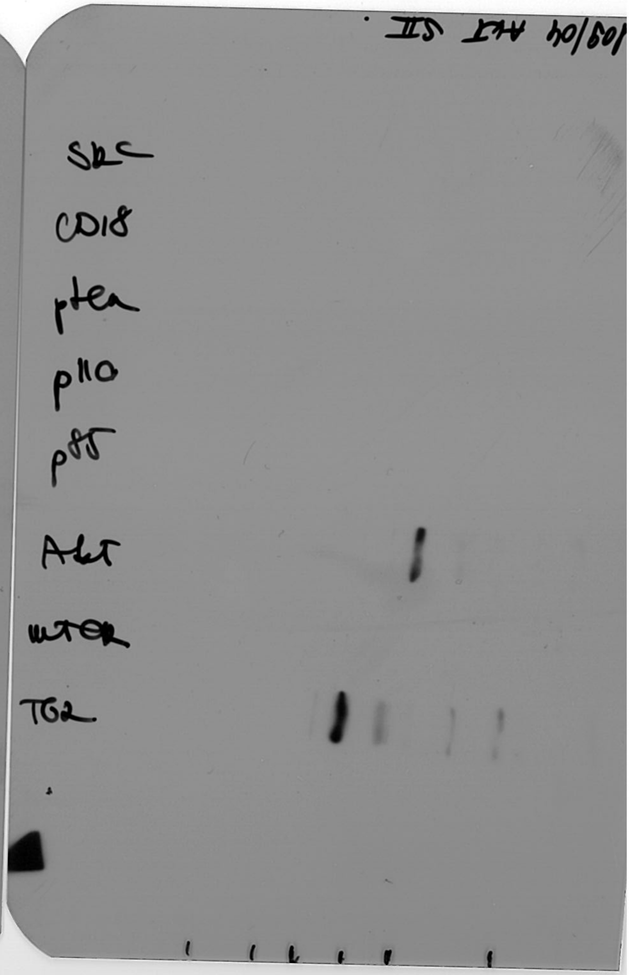
AKT SII
SRC
CD18
pten
p110
p85
Akt
mTOR
TG2

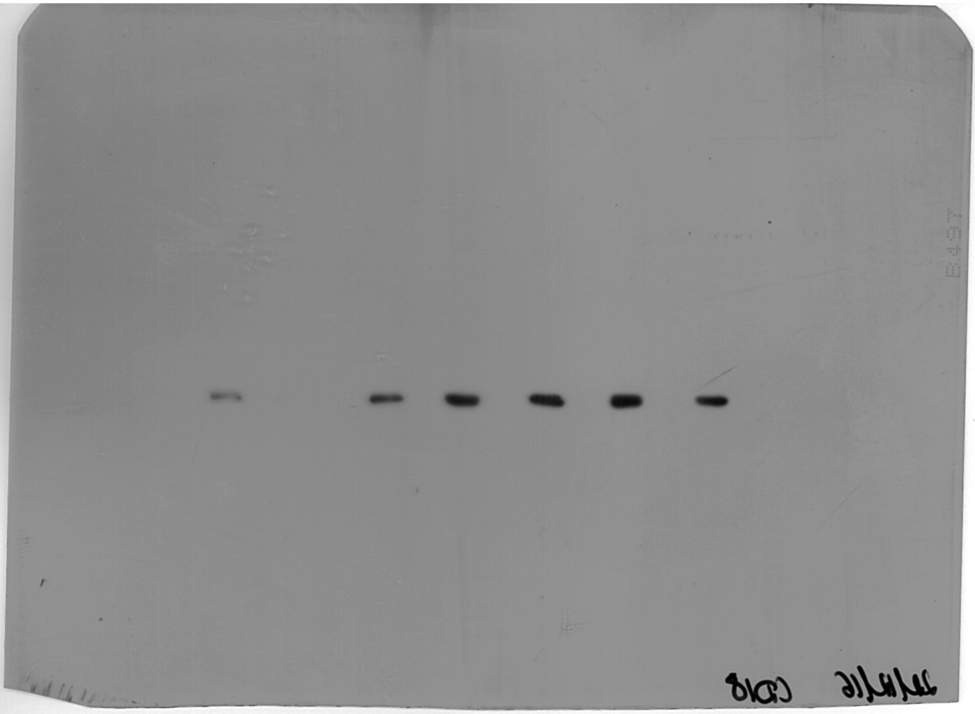

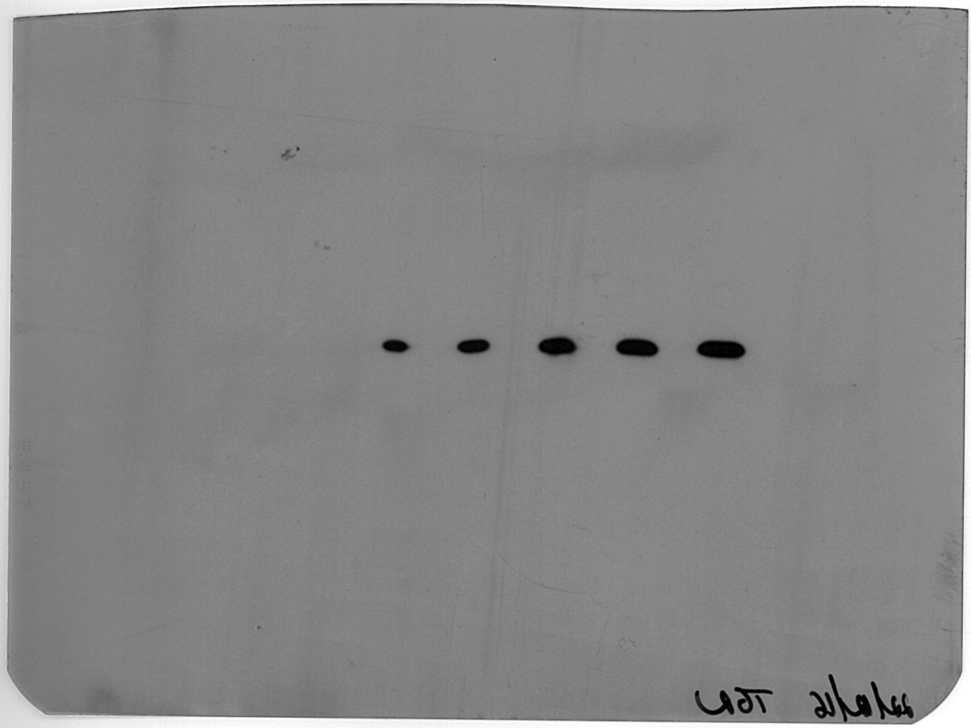

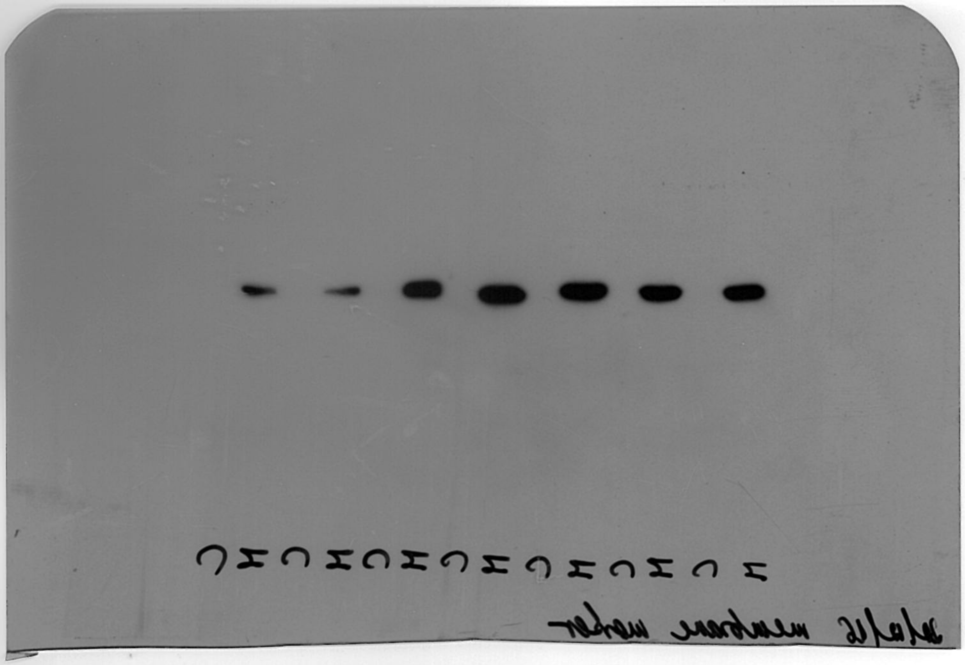

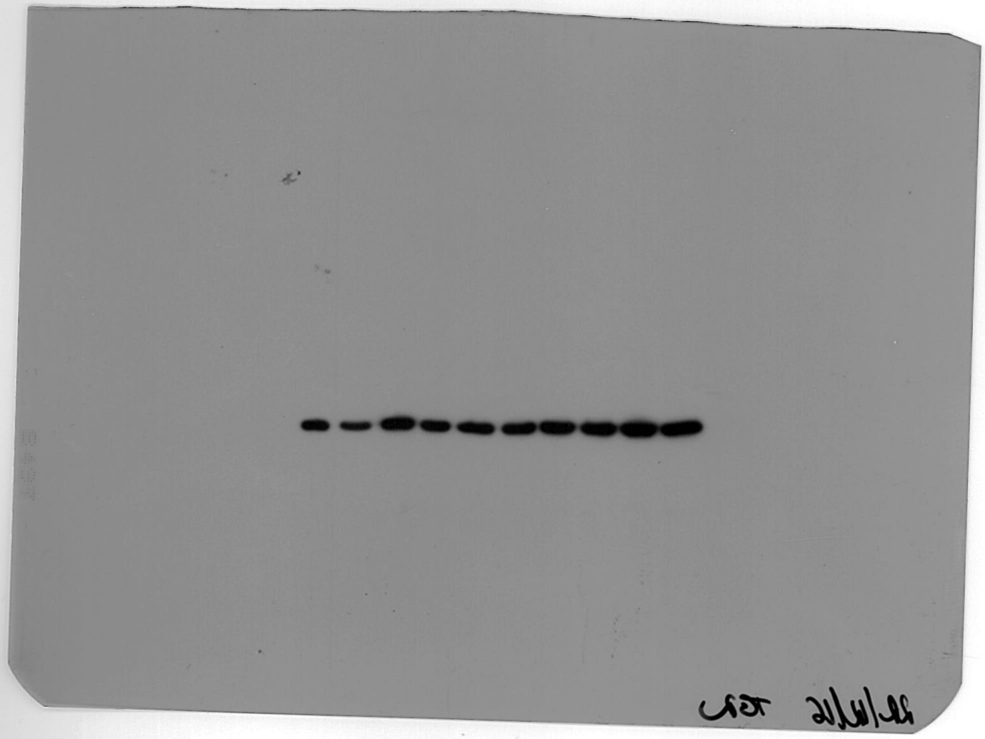

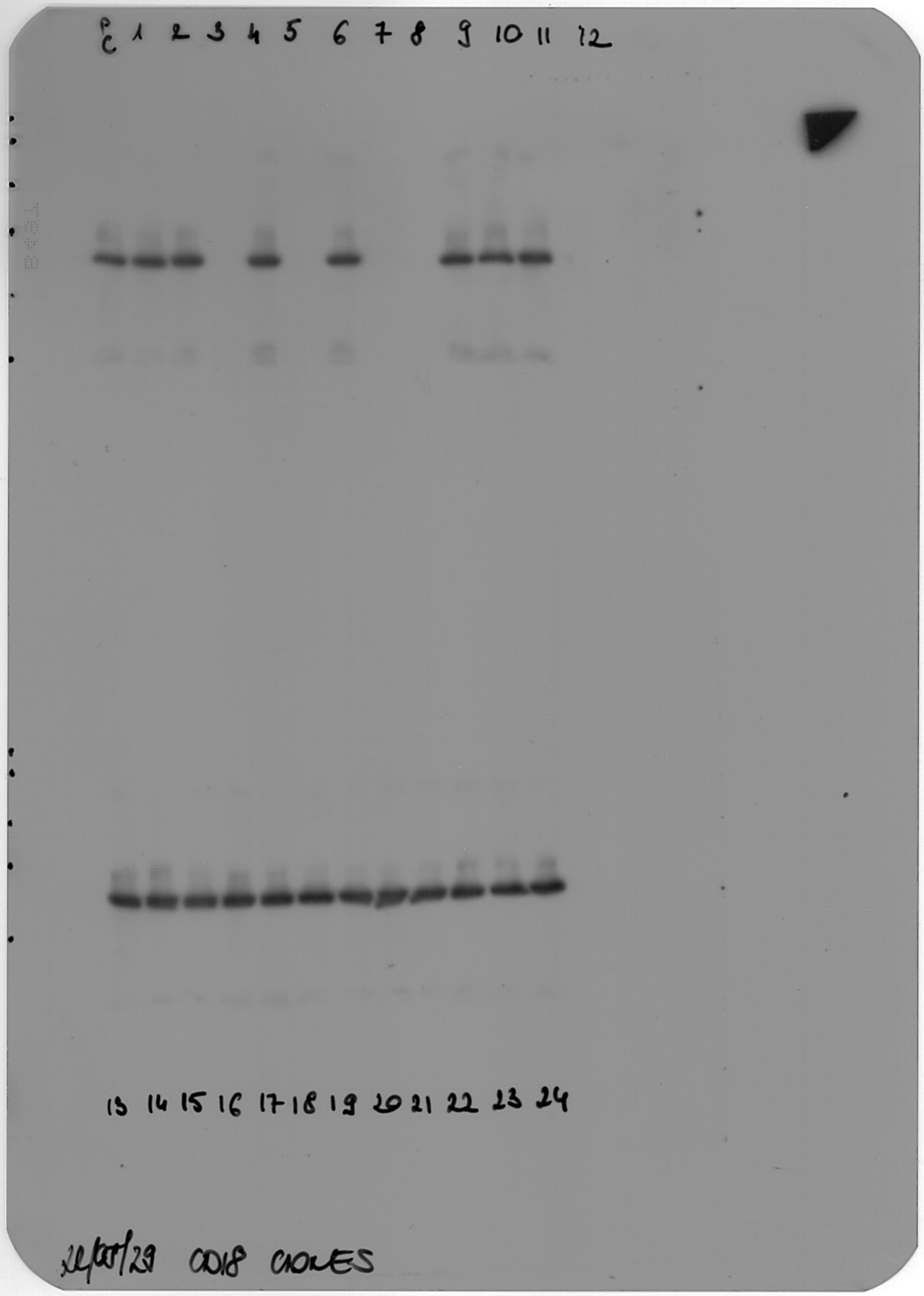
PC 1 2 3 4 5 6 7 8 9 10 11 12
13 14 15 16 17 18 19 20 21 22 23 24
CD18 CLONES

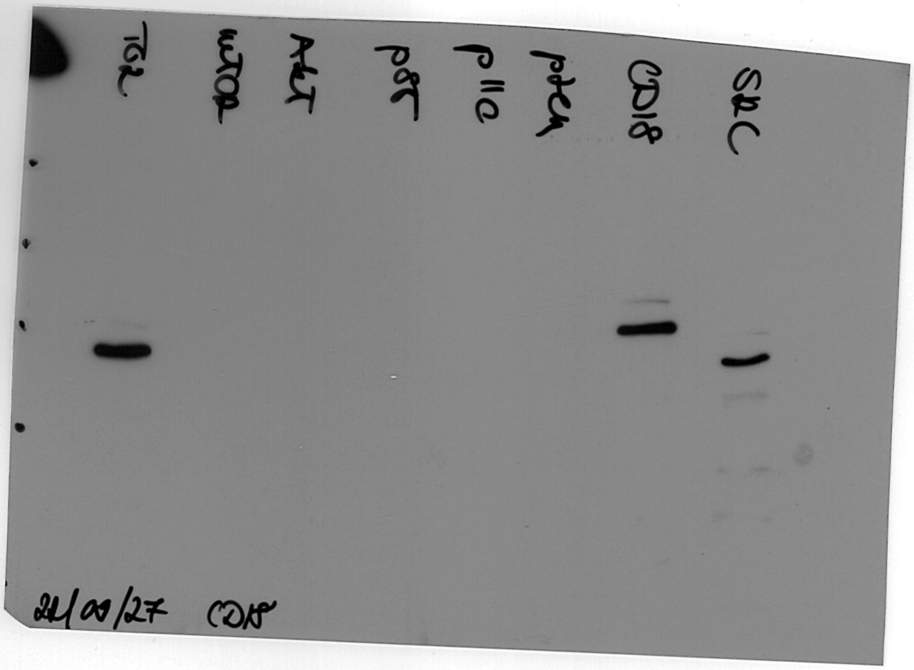
mTOR
AKT
p110
CD18
SRC
22/09/27 CD18

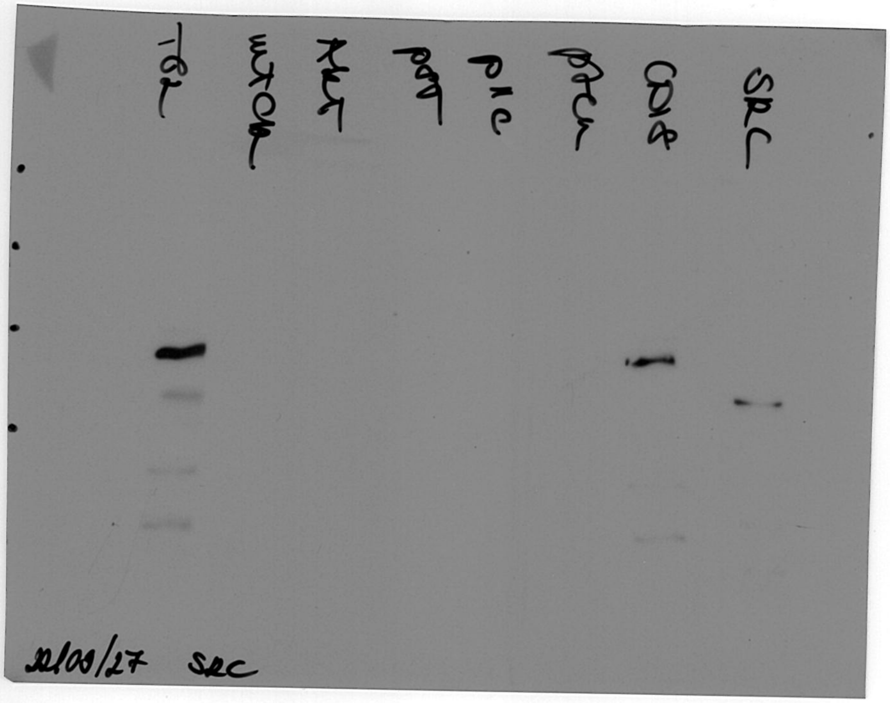
mTOR
AKT
p110
CD18
SRC
22/09/27 SRC

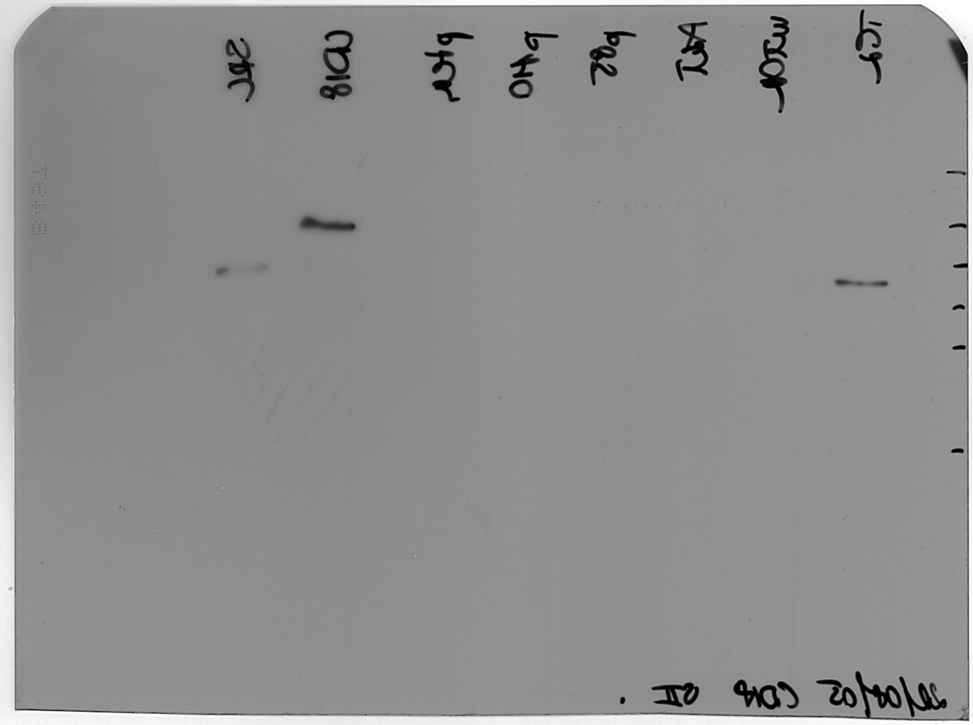

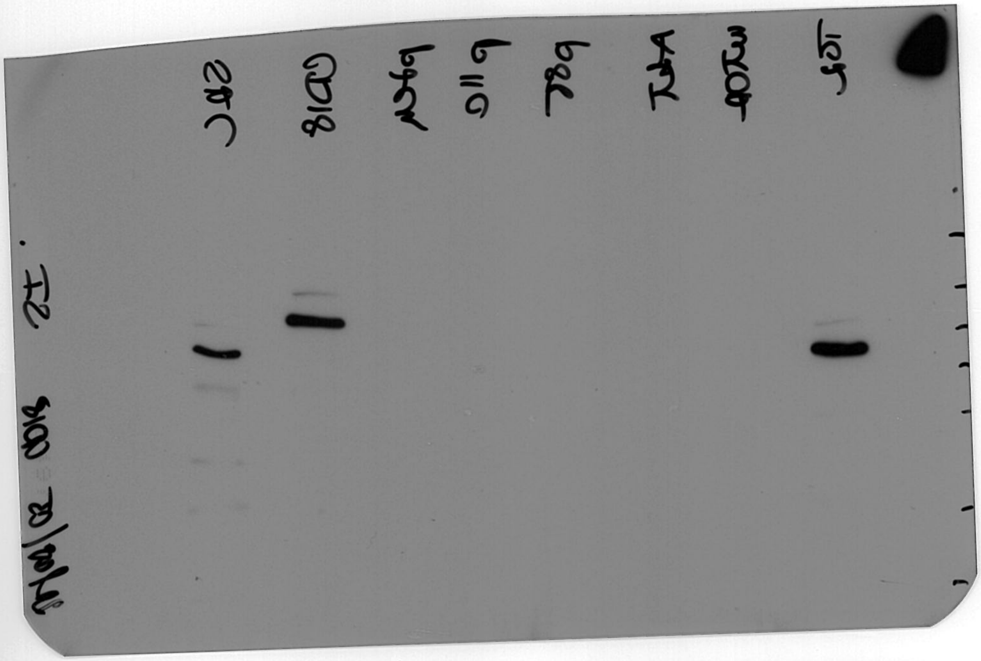

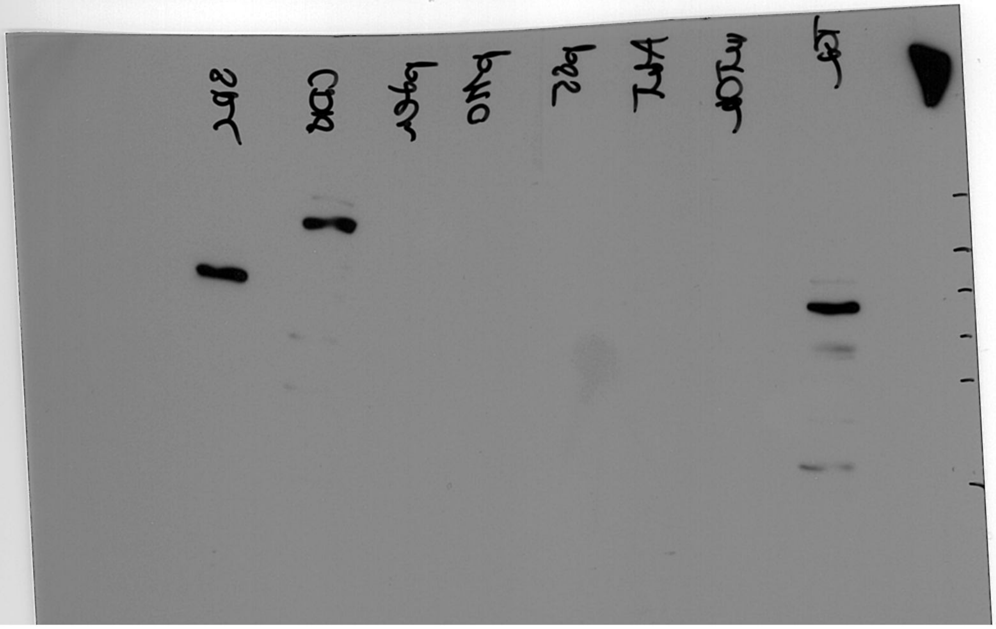

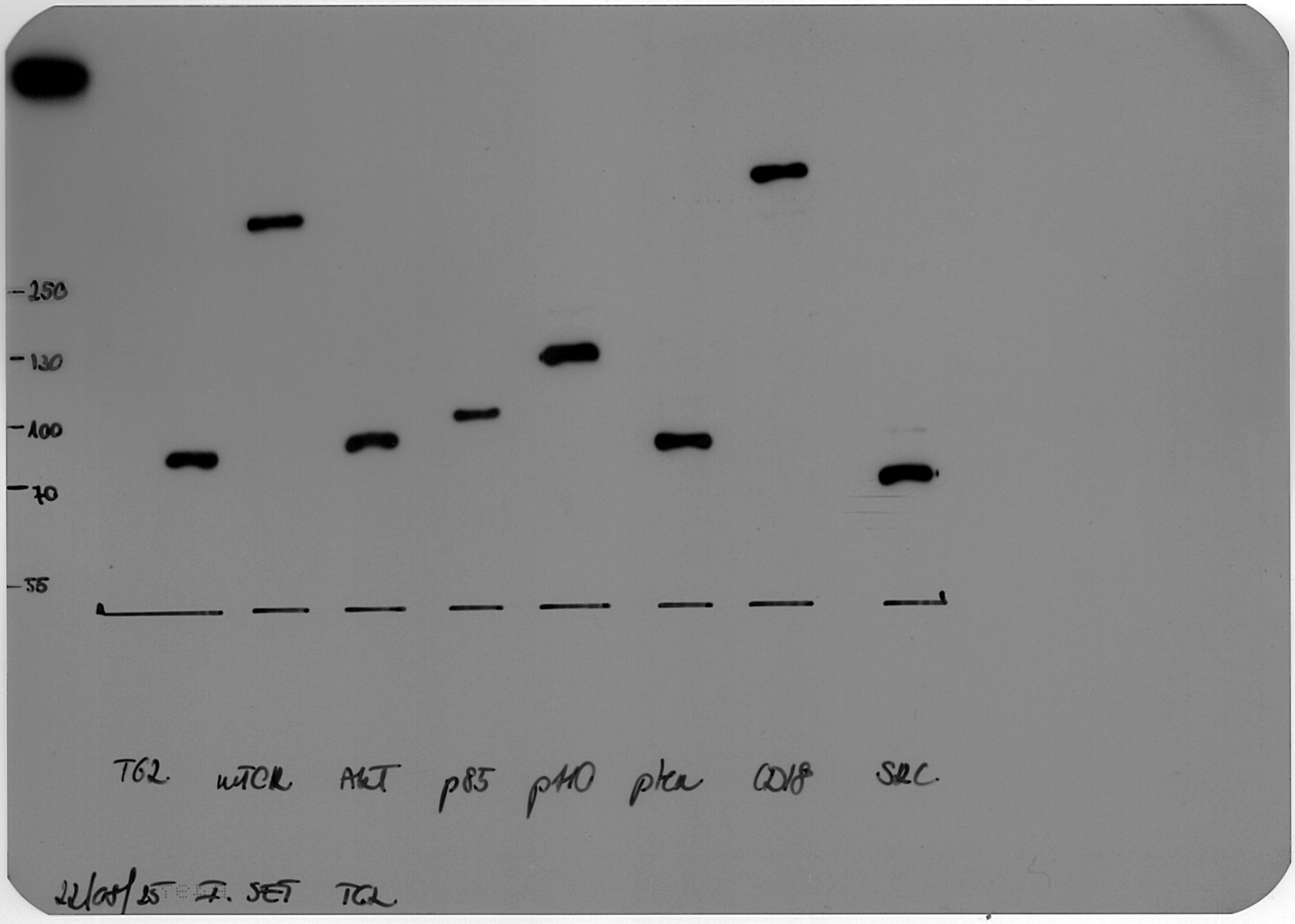
250
130
100
70
55
TG2
mTOR
AKT
p85
p110
pten
CD18
SRC.
22/08/25 I. SET TG2

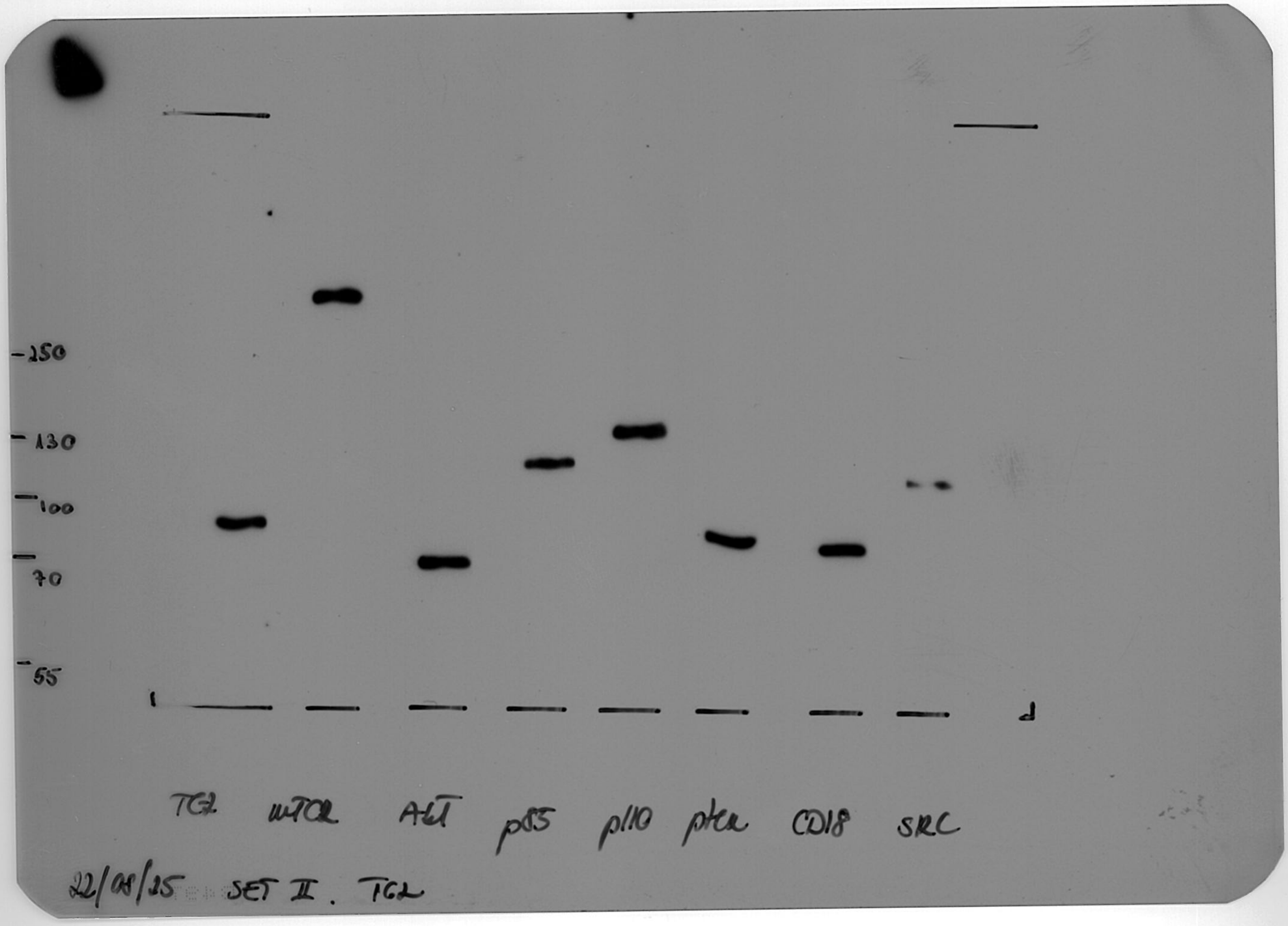
250
130
100
70
55
TG2
mTOR
AKT
p85
p110
pten
CD18
SRC
22/08/25 SET II. TG2

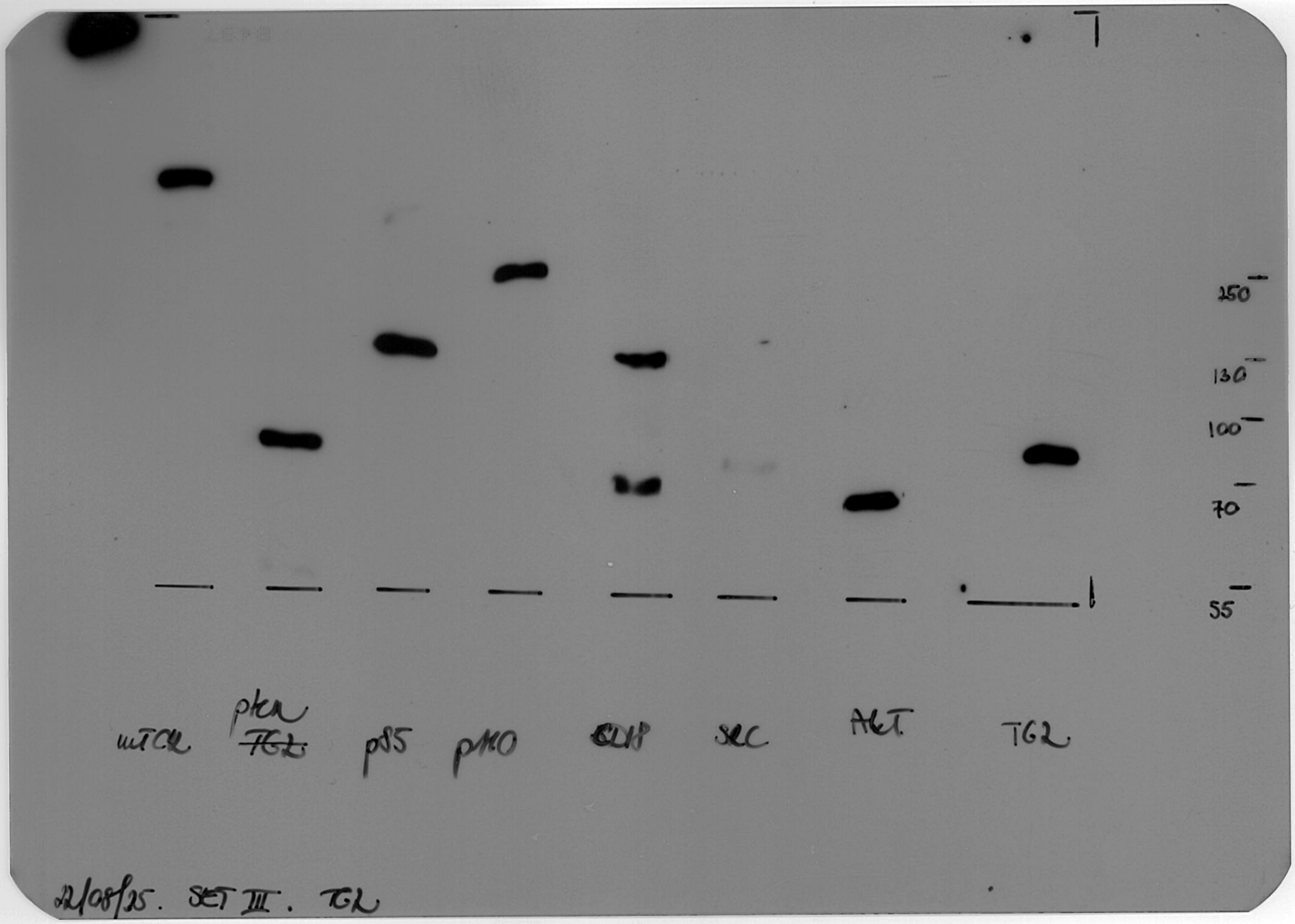
mTOR
pten
p85
p110
SRC
AKT.
TG2
250
130
100
70
55
22/08/25. SET III. TG2

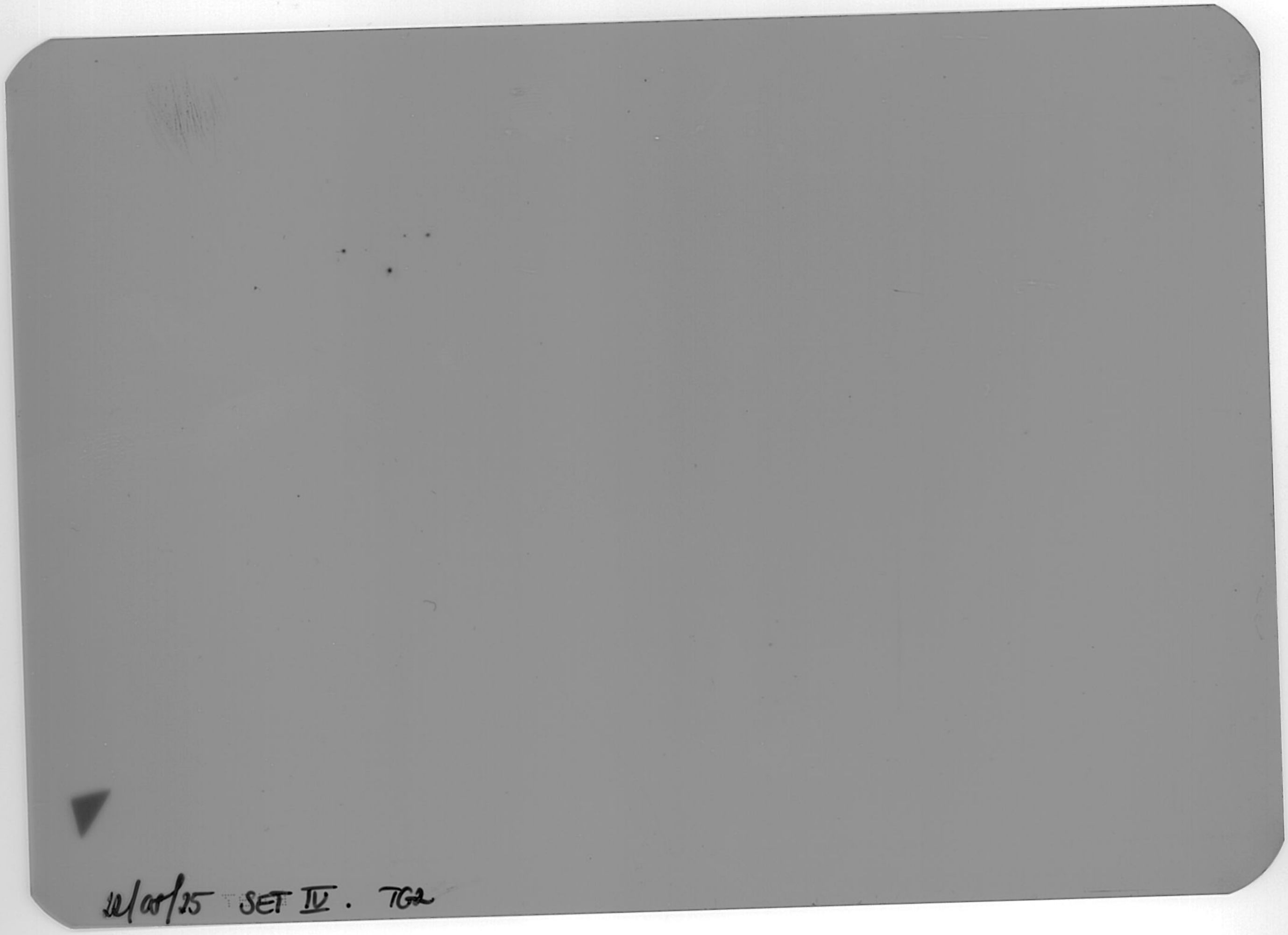
22/08/25 SET IV. TG2

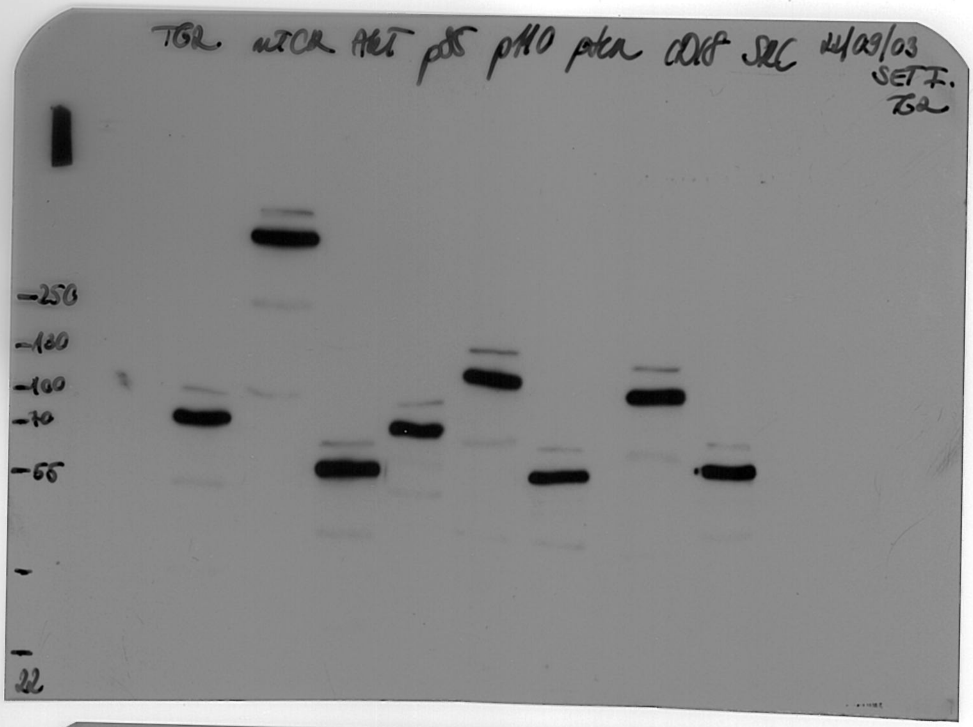
TGR mTOR AKT p85 p110 pten CD18 SRC
22/09/03
SET I.
TGR
250
130
100
70
55
22
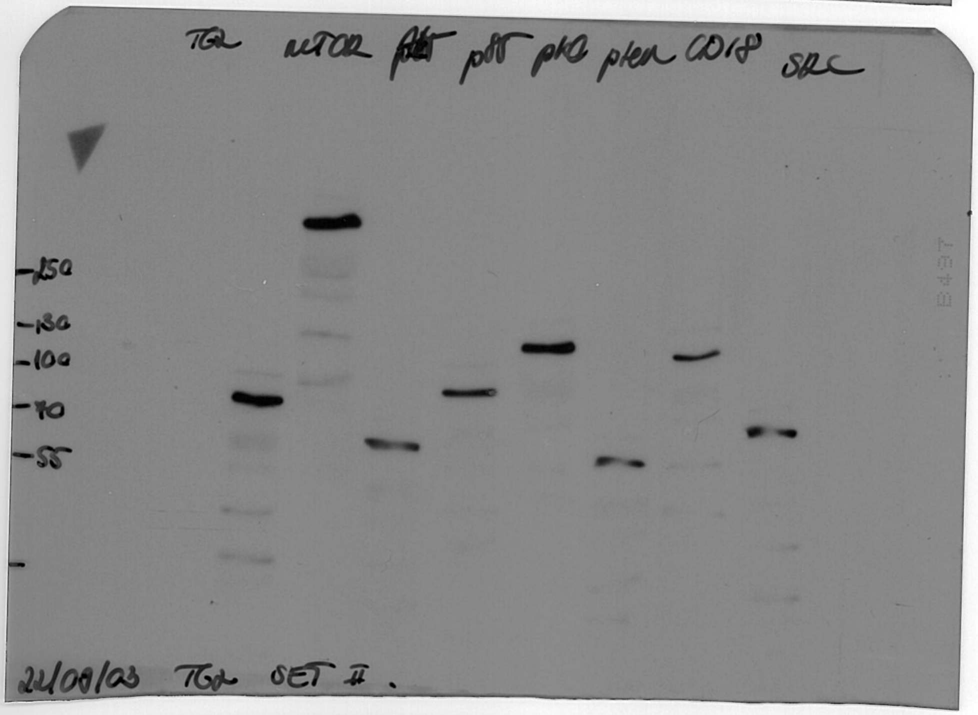
TGR mTOR Akt p85 p110 pten CD18 SRC
250
130
100
70
55
22/09/03 TGR SET II.
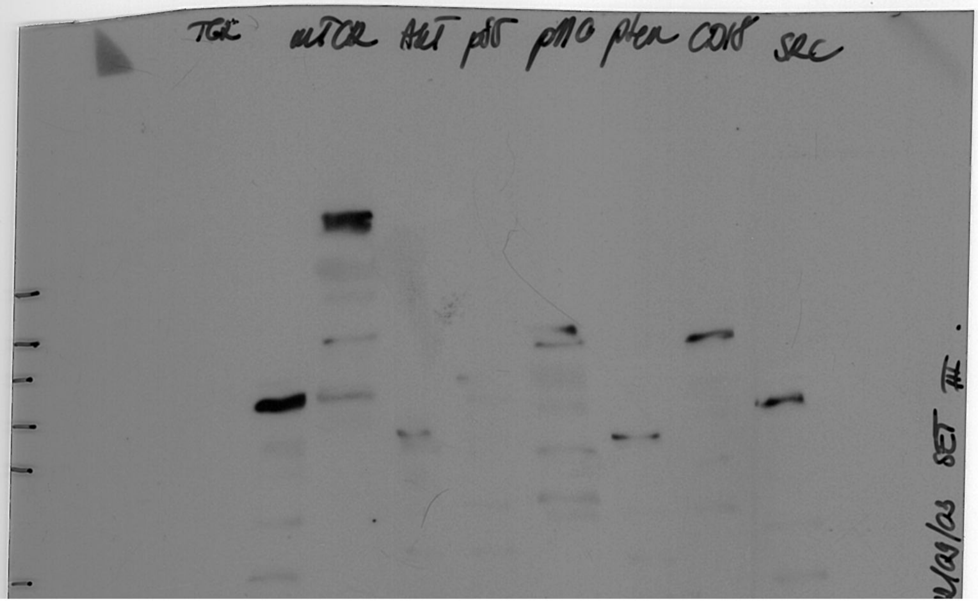
TGR mTOR AKT p85 p110 pten CD18 SRC
SET III.

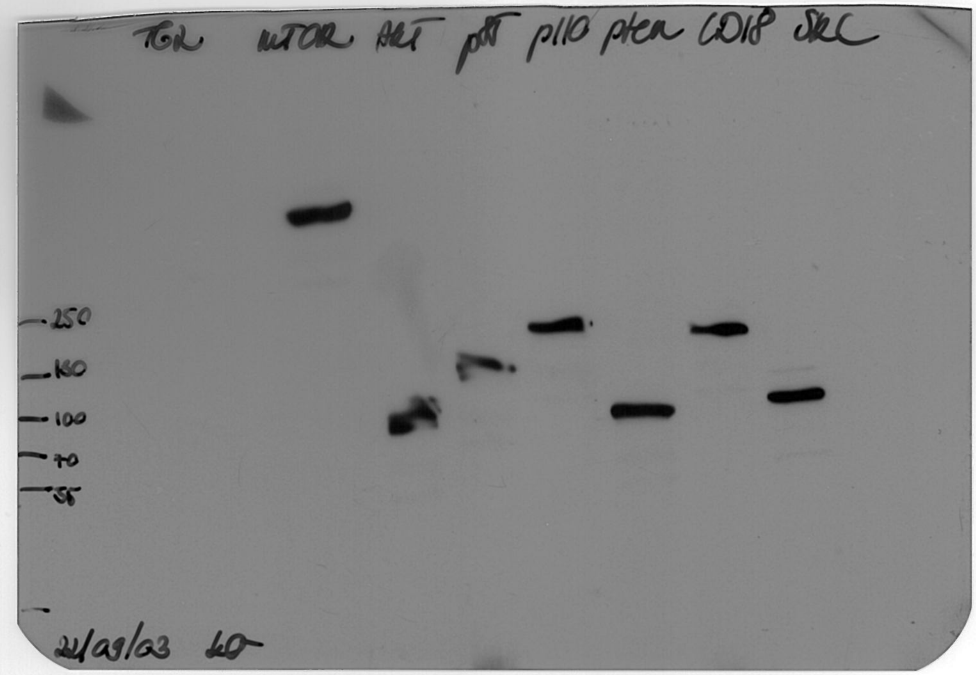
TGL mTOR AKT p85 p110 pten CD18 SRC
250
150
100
70
55
22/09/03 LO

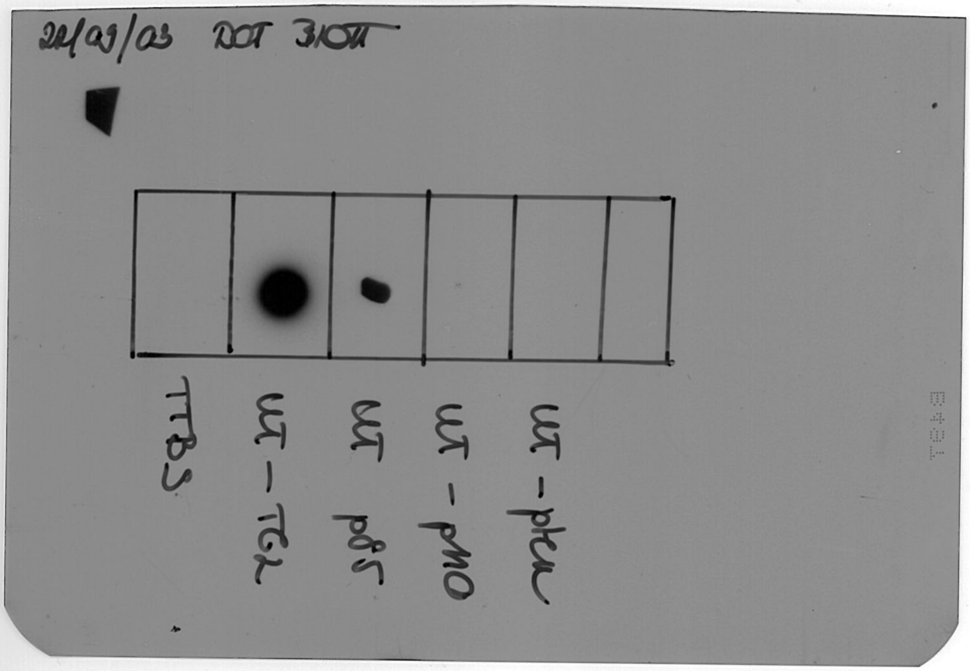
22/09/03 DOT BLOT
TTBS
WT - TGL
WT p85
WT - p110
WT - pten

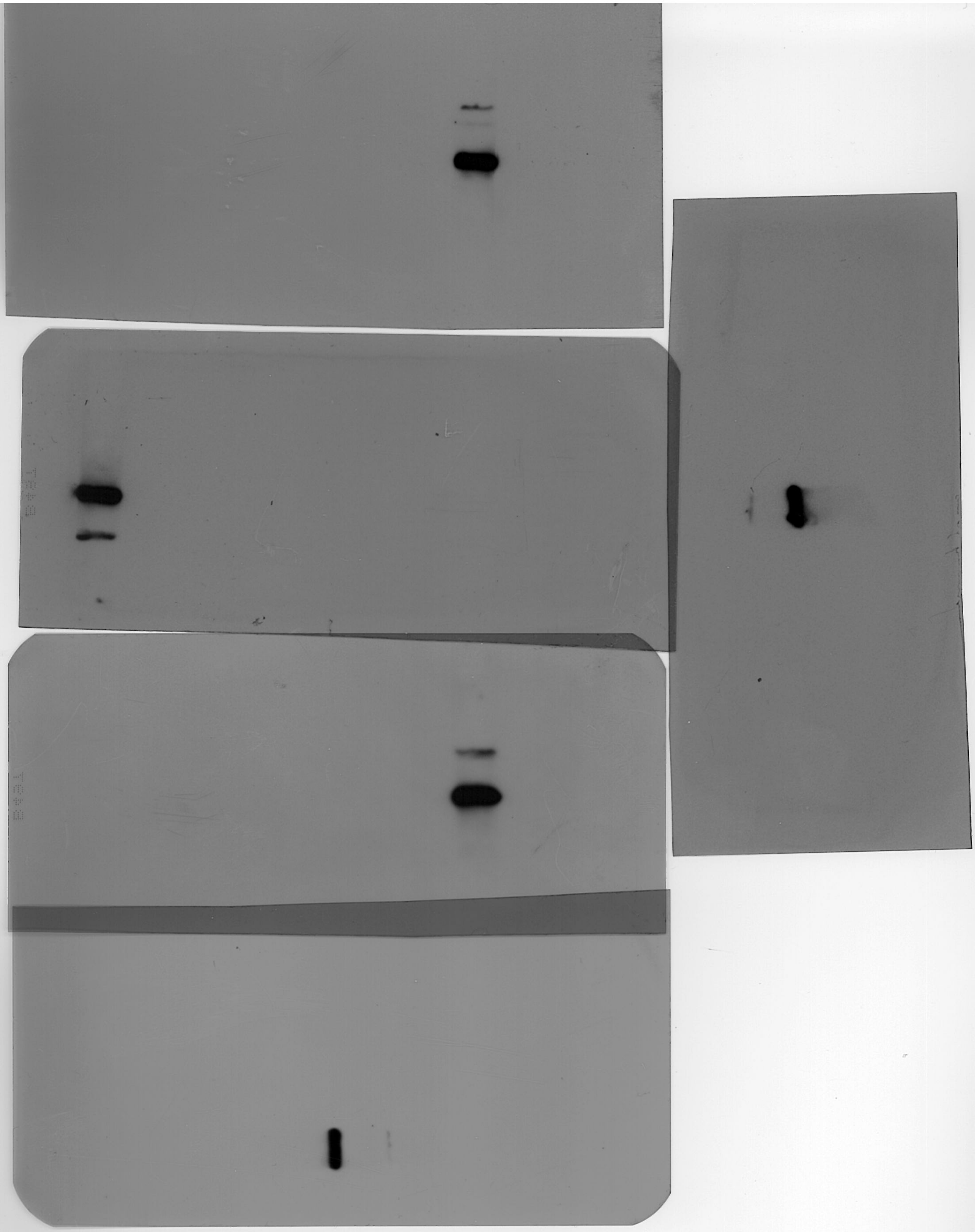

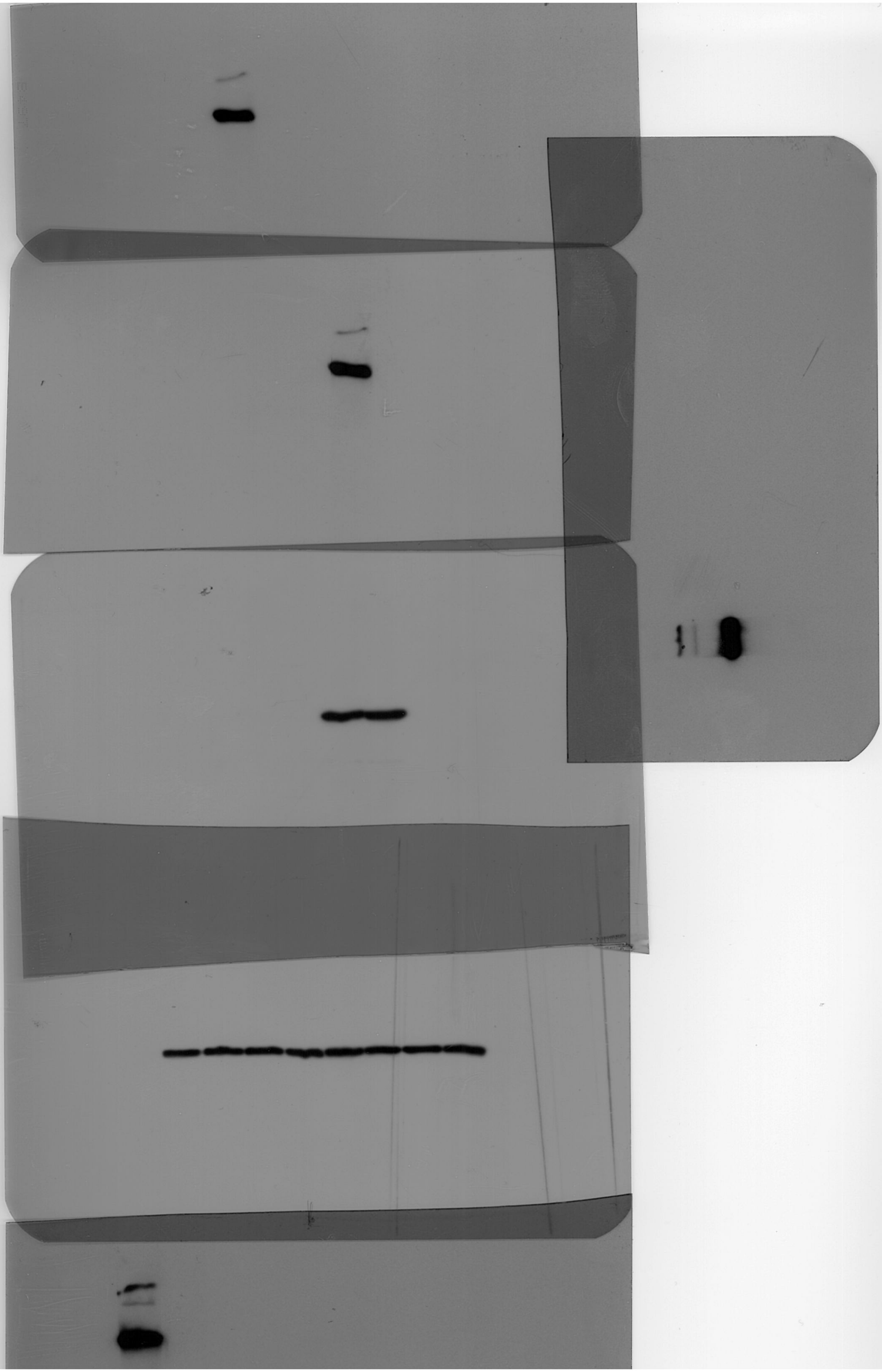

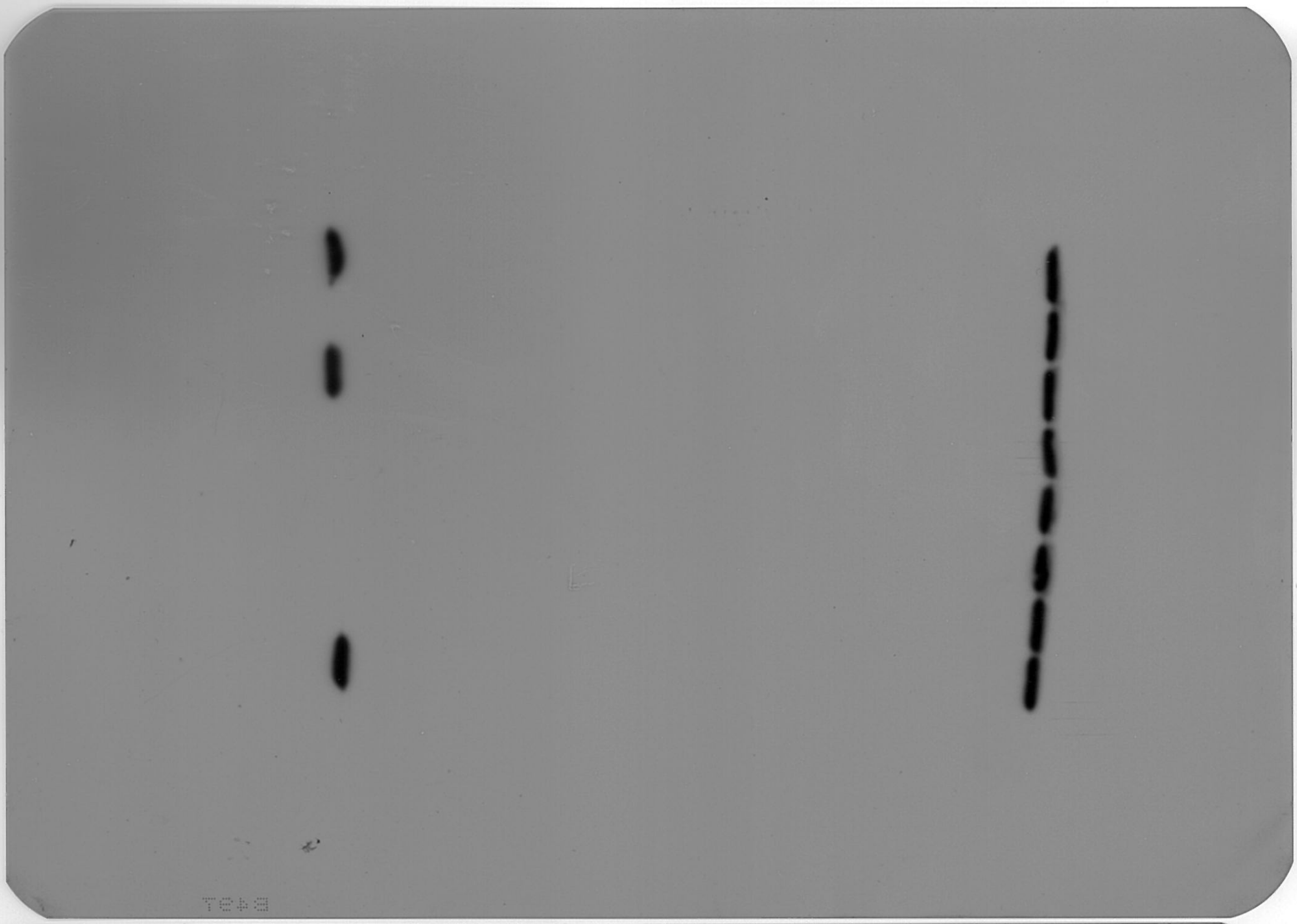

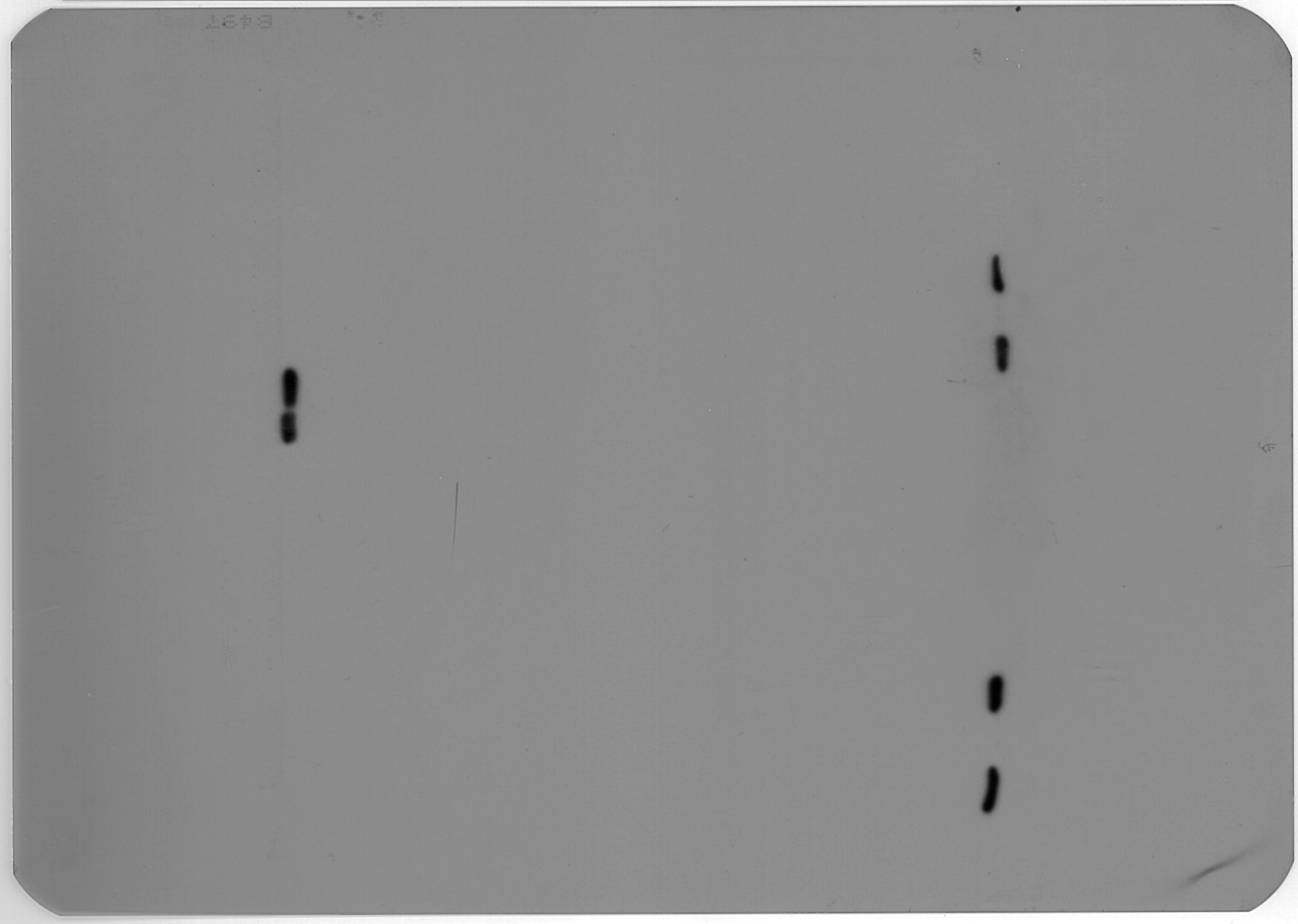

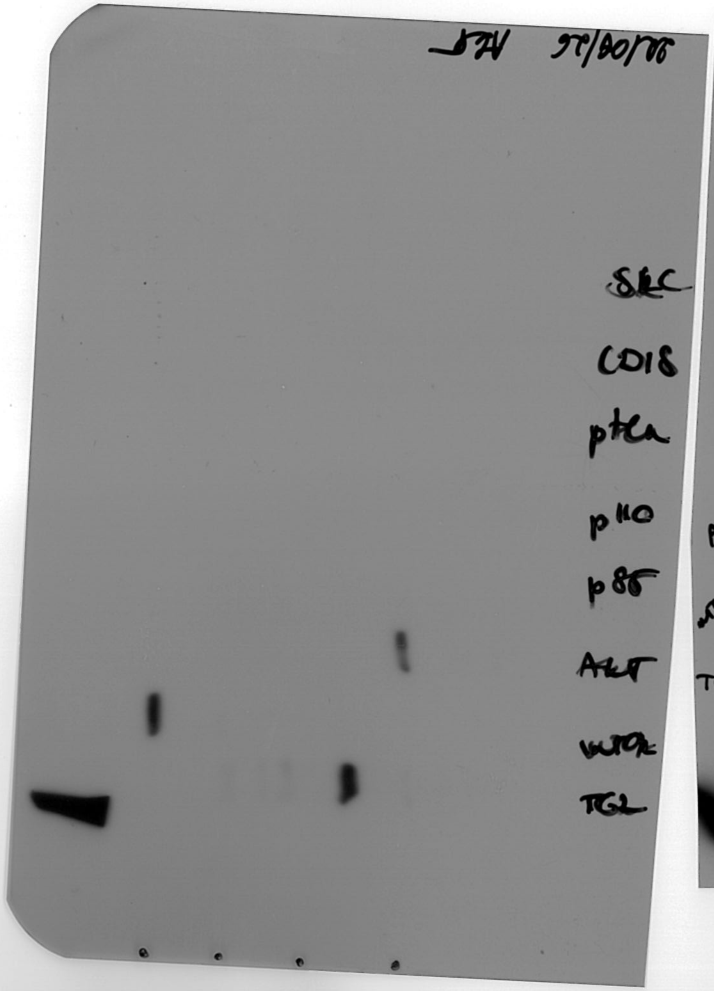
SRC
CD18
pten
p110
p85
Akt
TCL

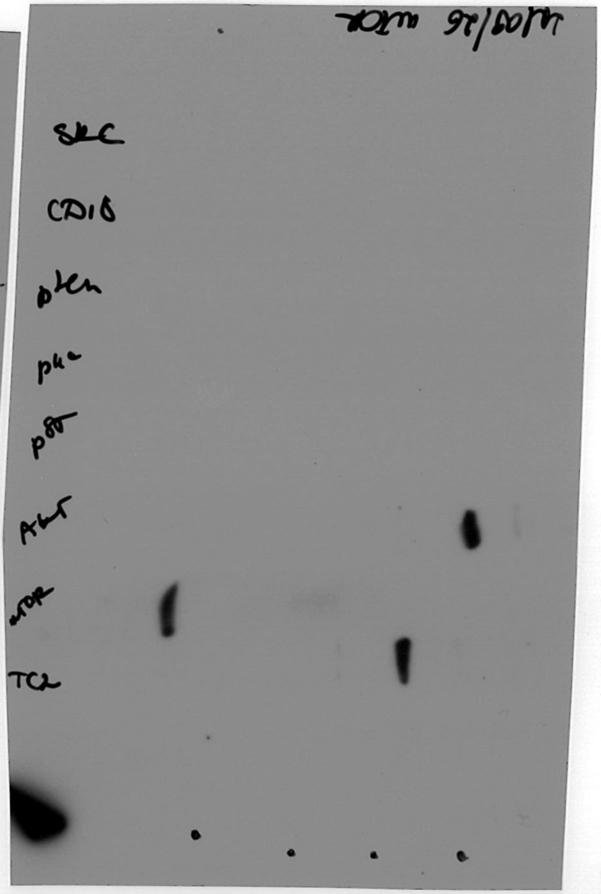
SRC
CD18
pten
p85
Akt
TCL

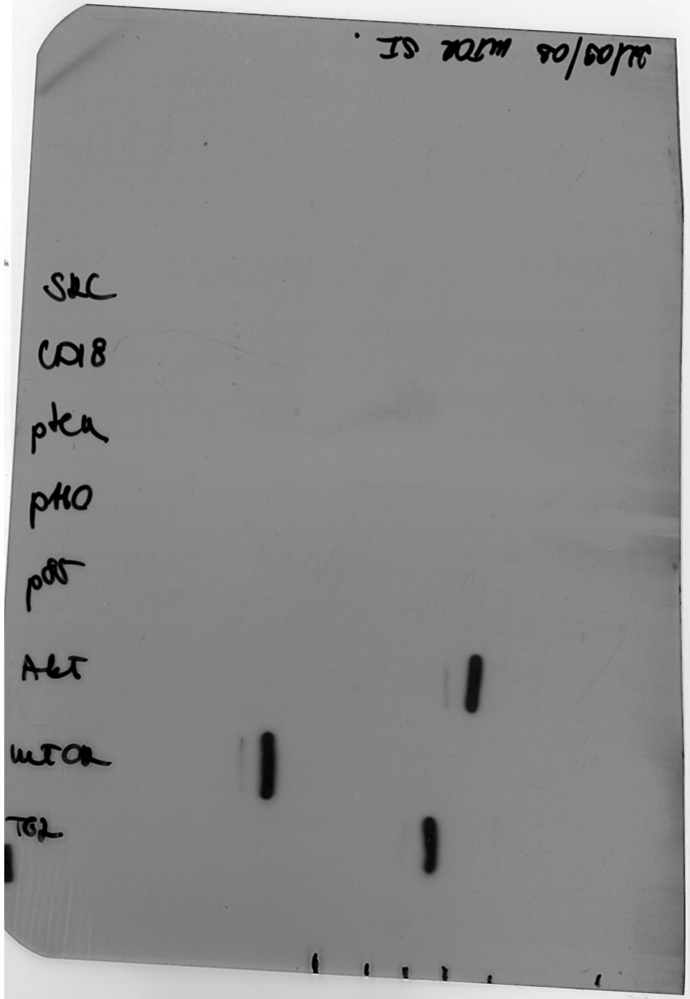
22/09/03 mTOR SI
SRC
CD18
pten
p110
p85
Akt
mTOR
TG2

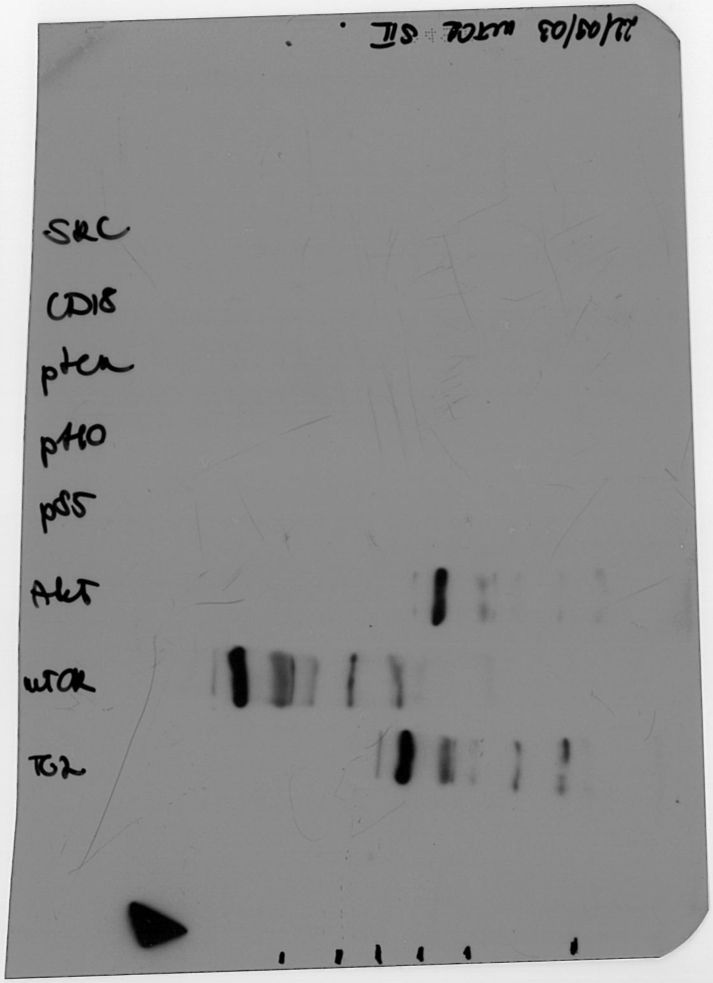
22/09/03 mTOR SII
SRC
CD18
pten
p110
p85
Akt
mTOR
TG2

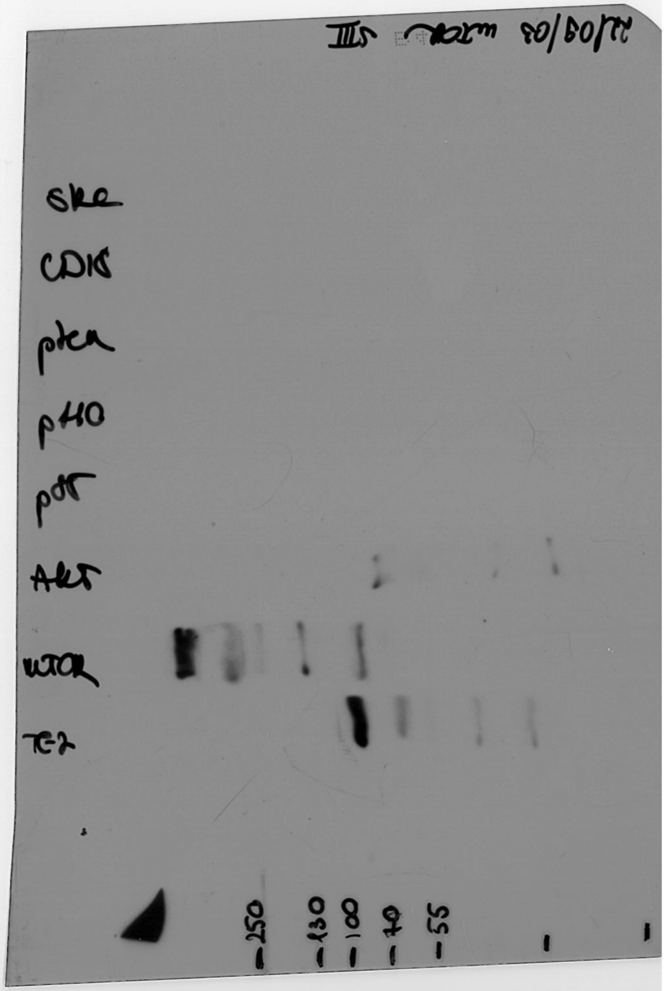
22/09/03 mTOR SIII
SRC
CD18
pten
p110
p85
Akt
mTOR
TG2
250
130
100
70
55

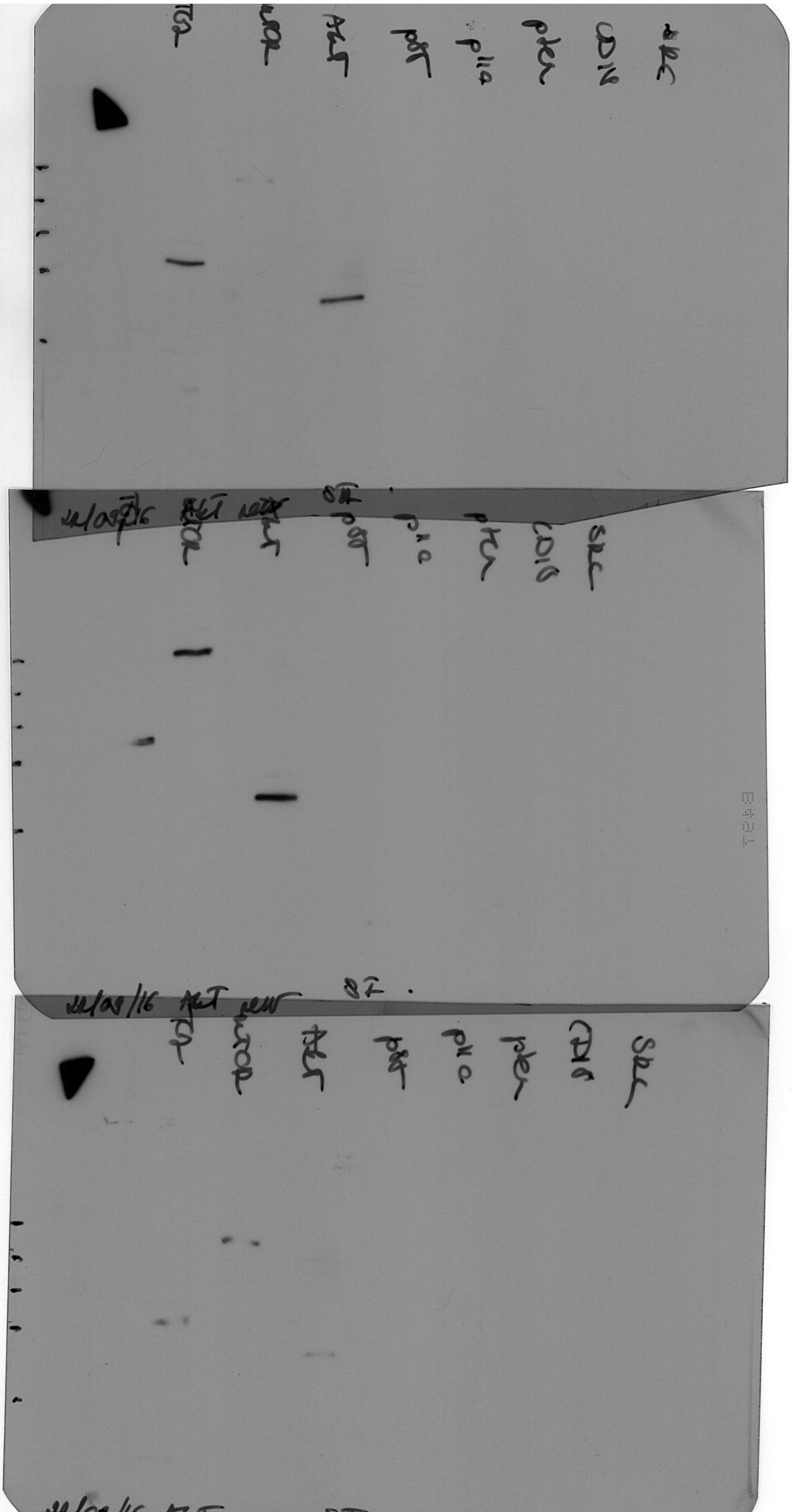
22/08/16 AKT new

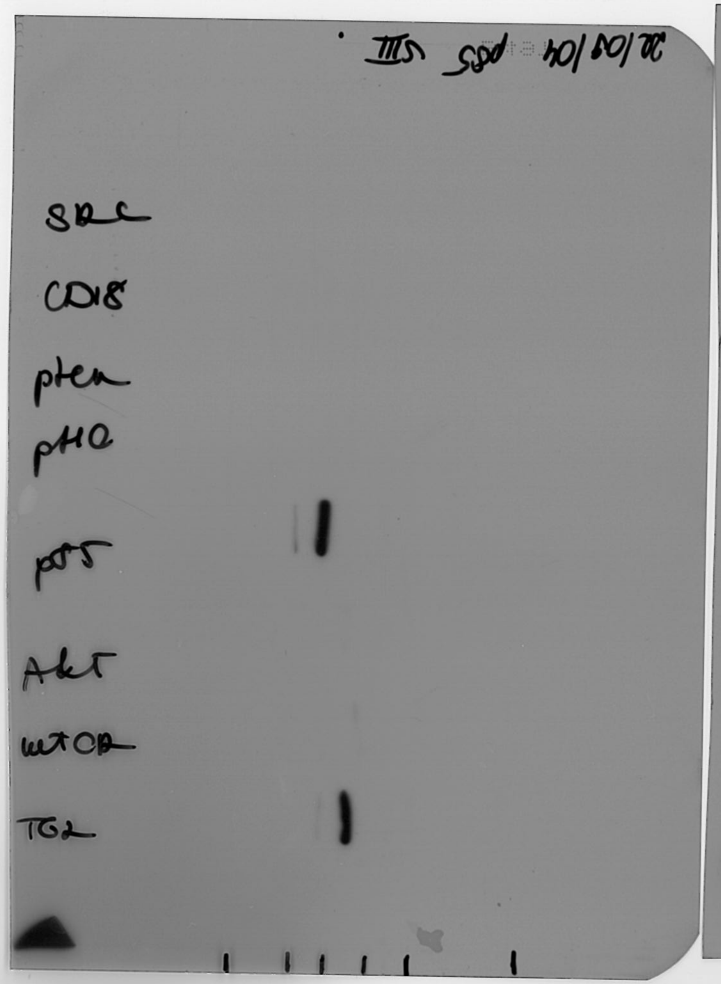
SRC
CD18
pten
p110
p85
Akt
mTOR
TG2

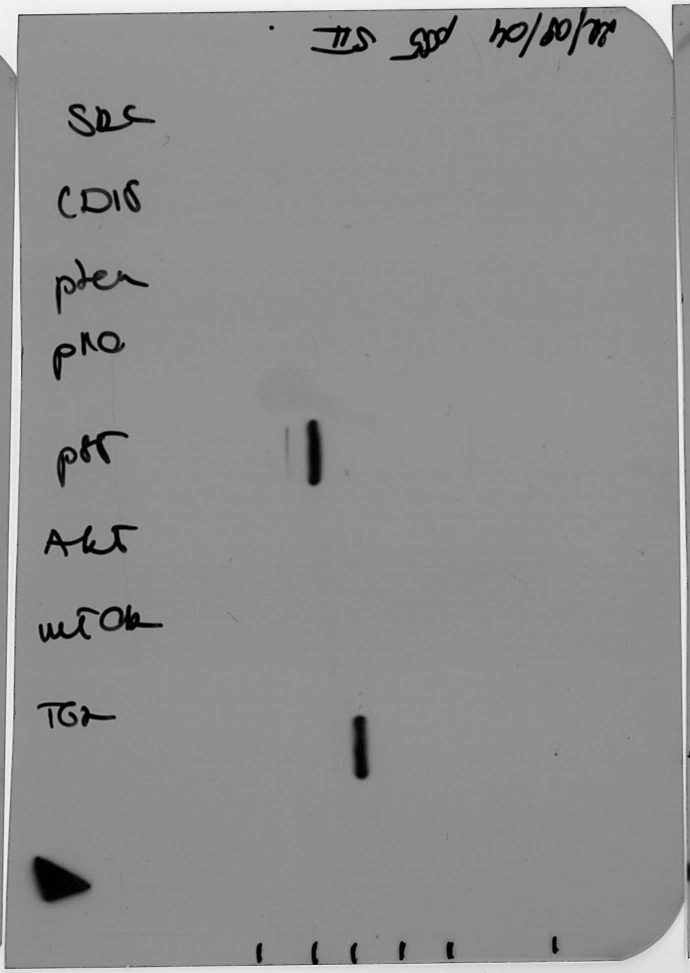
SRC
CD18
pten
p110
p85
Akt
mTOR
TG2

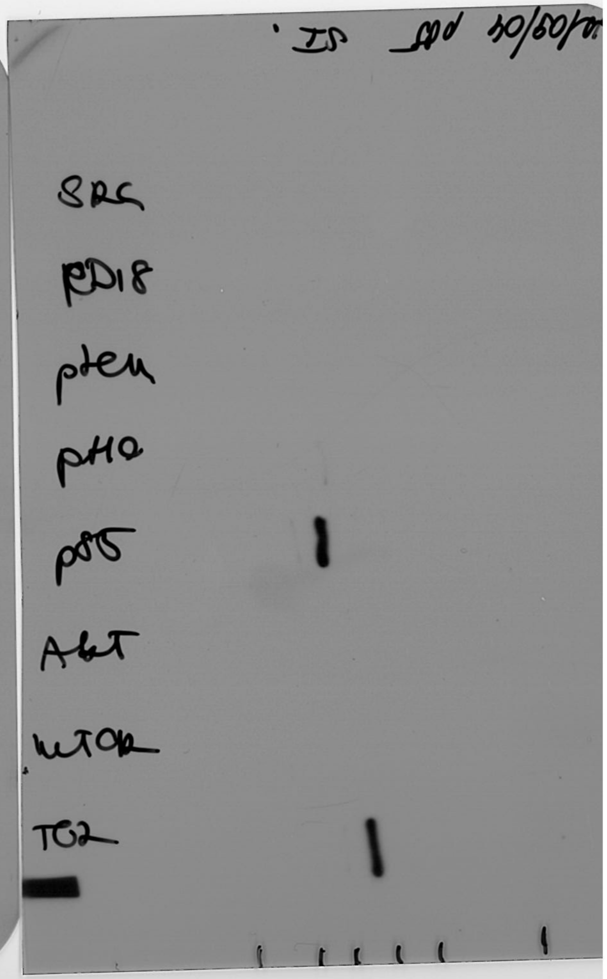
SRC
CD18
pten
p110
p85
Akt
mTOR
TG2

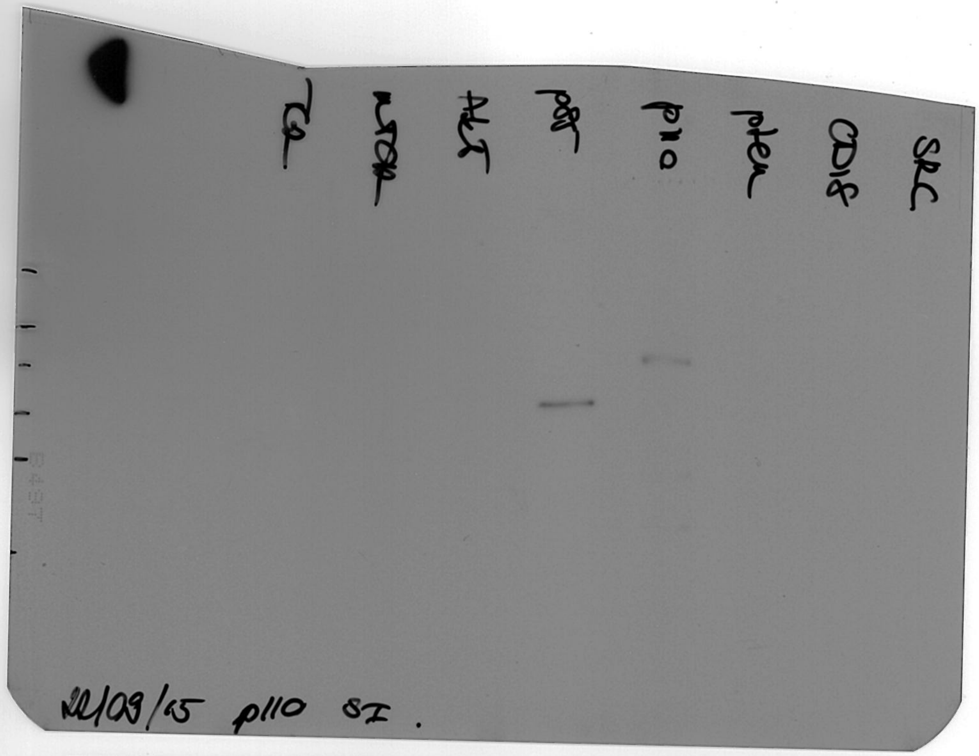
TCL
mTOR
AKT
p85
p110
pten
CD19
SRC
22/09/15 p110 SI.

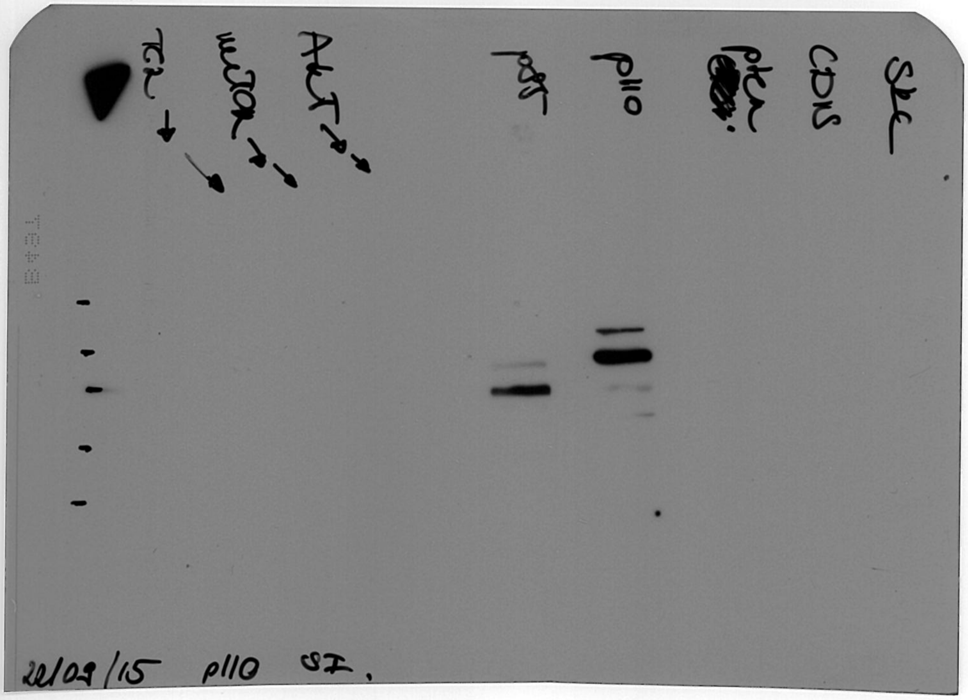
TCL
mTOR
AKT
p85
p110
pten
CD19
SRC
22/09/15 p110 SI.

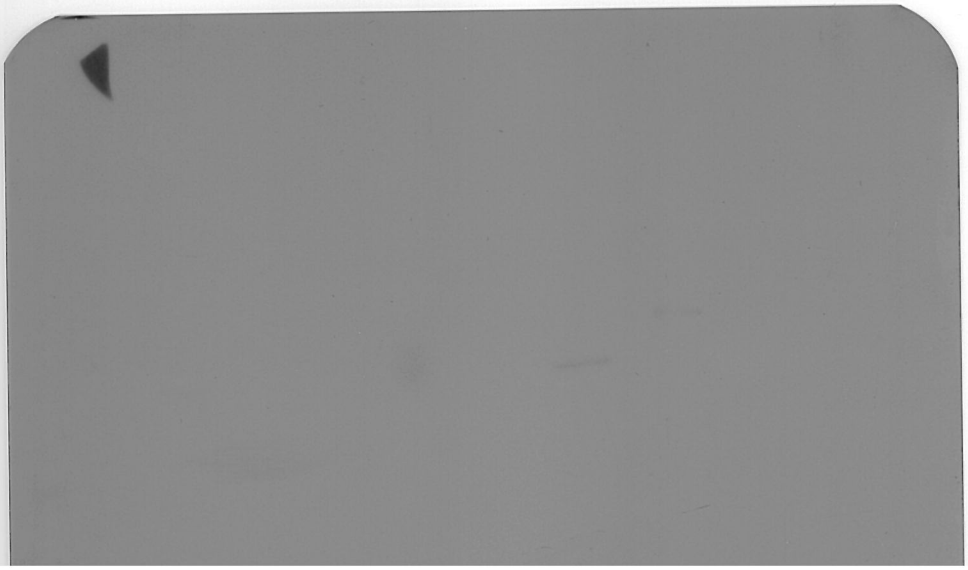

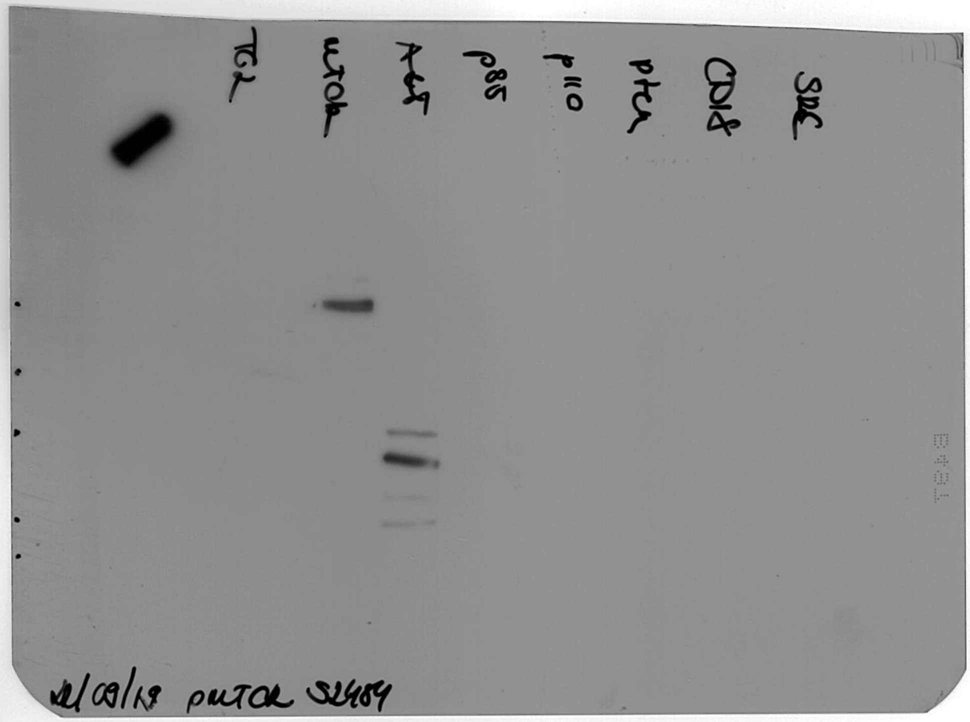
mTOR
Akt
p85
p110
pten
pmTOR

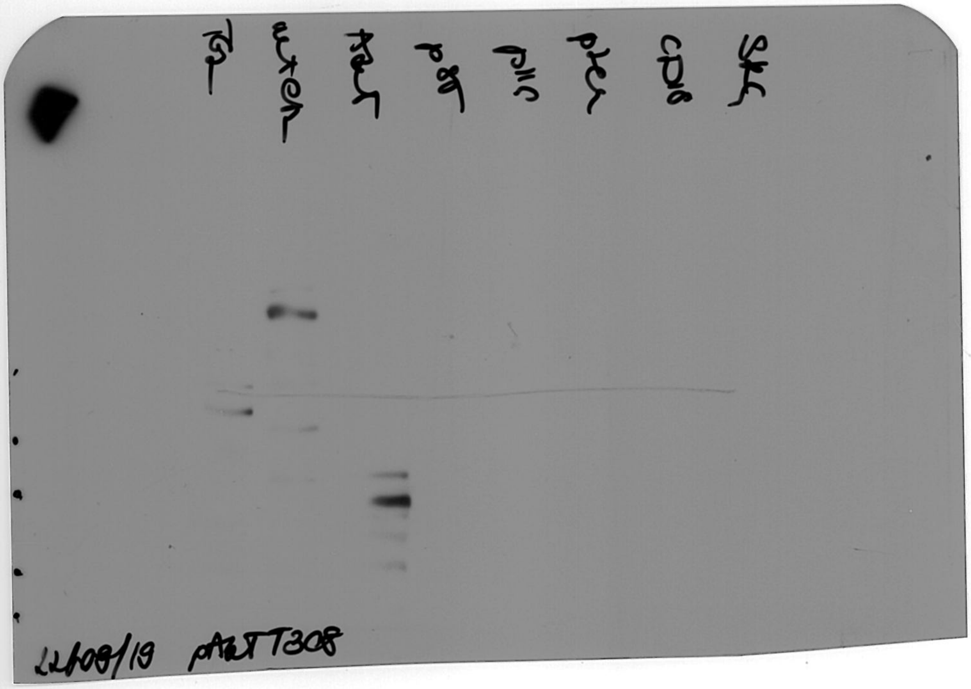
mTOR
pten
22/09/19 pAKT T308

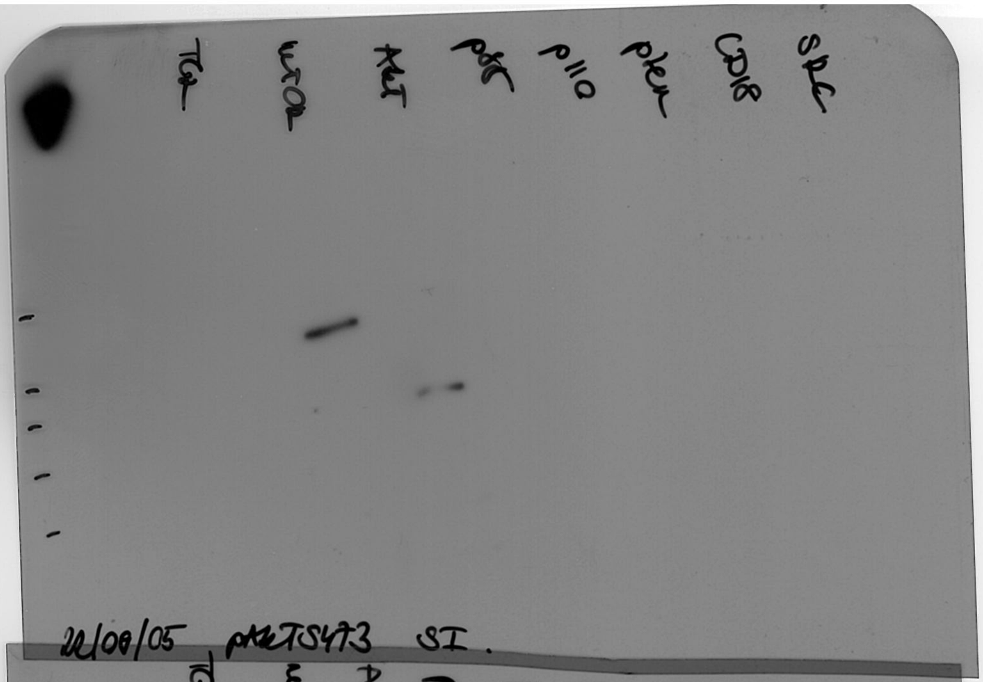
mTOR
AKT
p110
pten
22/09/05 pAKT S473 SI.
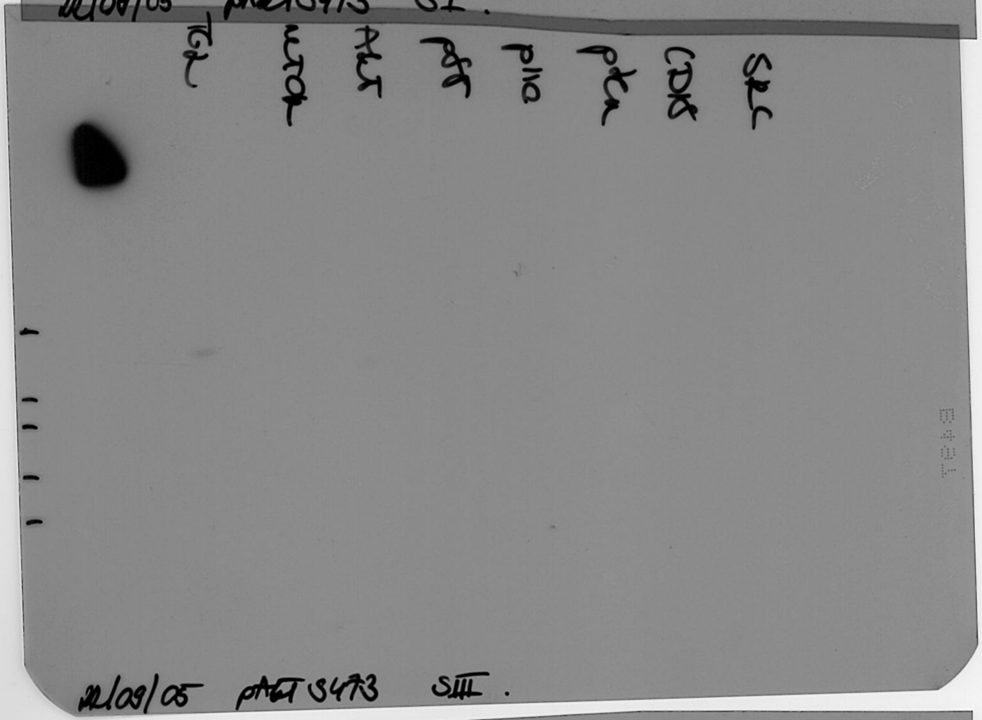
mTOR
AKT
p110
pten
22/09/05 pAKT S473 SII.
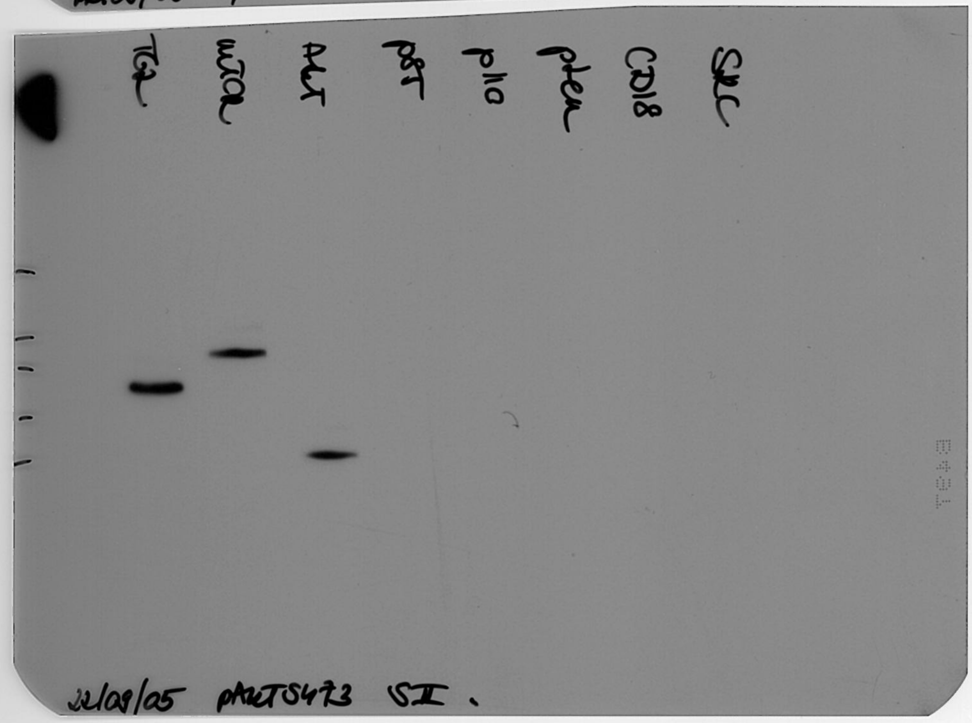
mTOR
AKT
p110
pten
22/09/05 pAKT S473 SI.

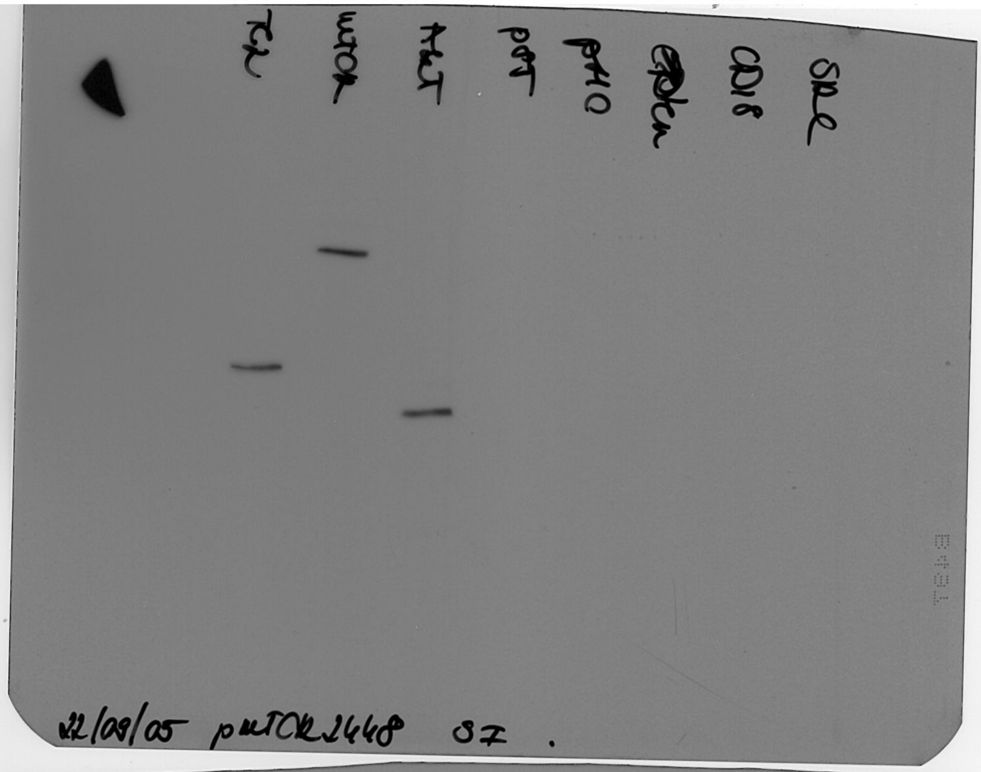
TCL
mTOR
AKT
p70
p110
pten
CD18
SRC
22/09/05 pmTOR2448 SI .

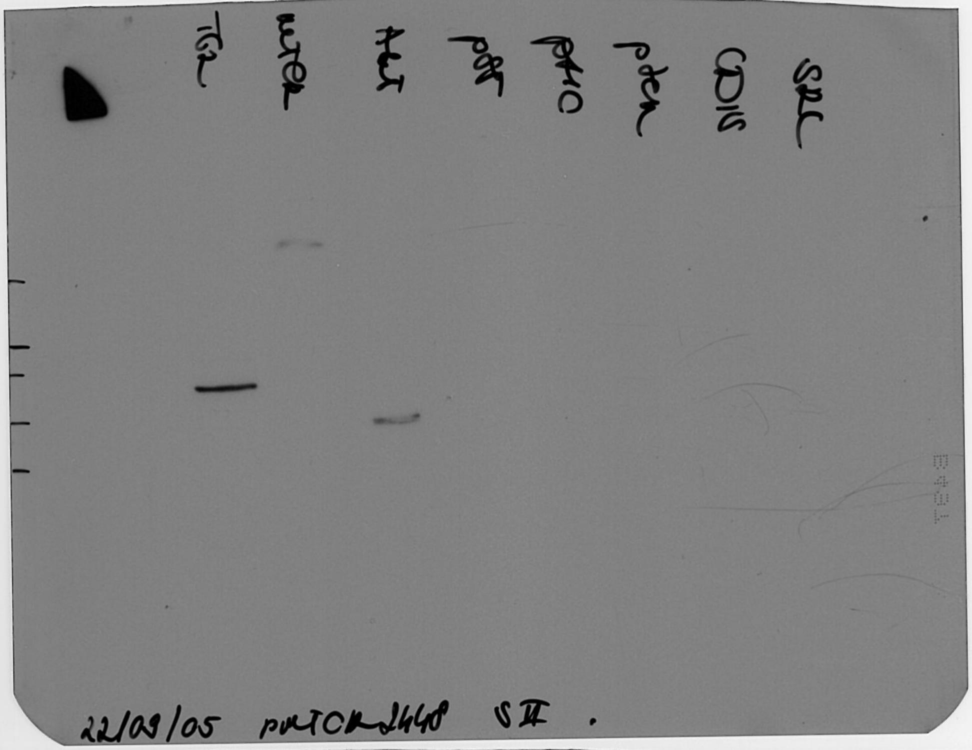
TCL
mTOR
AKT
p70
p110
pten
CD18
SRC
22/09/05 pmTOR2448 SII .

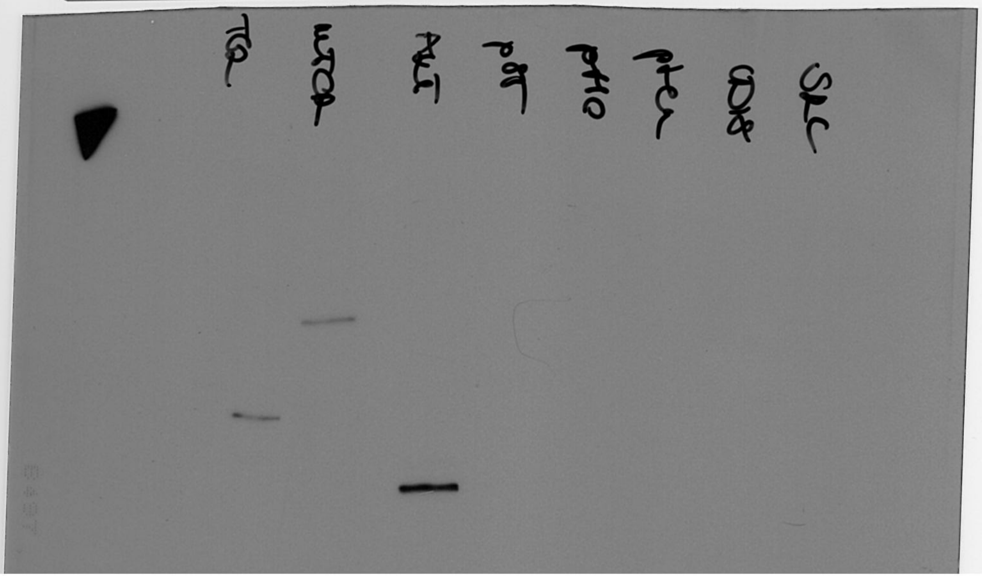
TCL
mTOR
AKT
p70
p110
pten
CD18
SRC

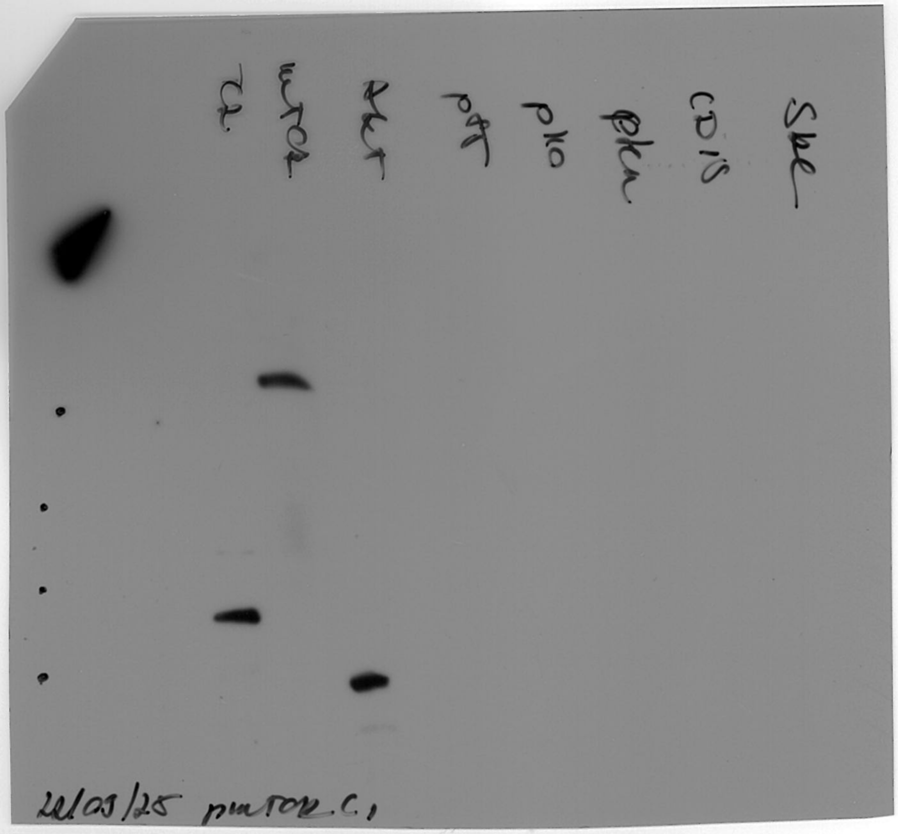
22/09/25 pmTOR.C1

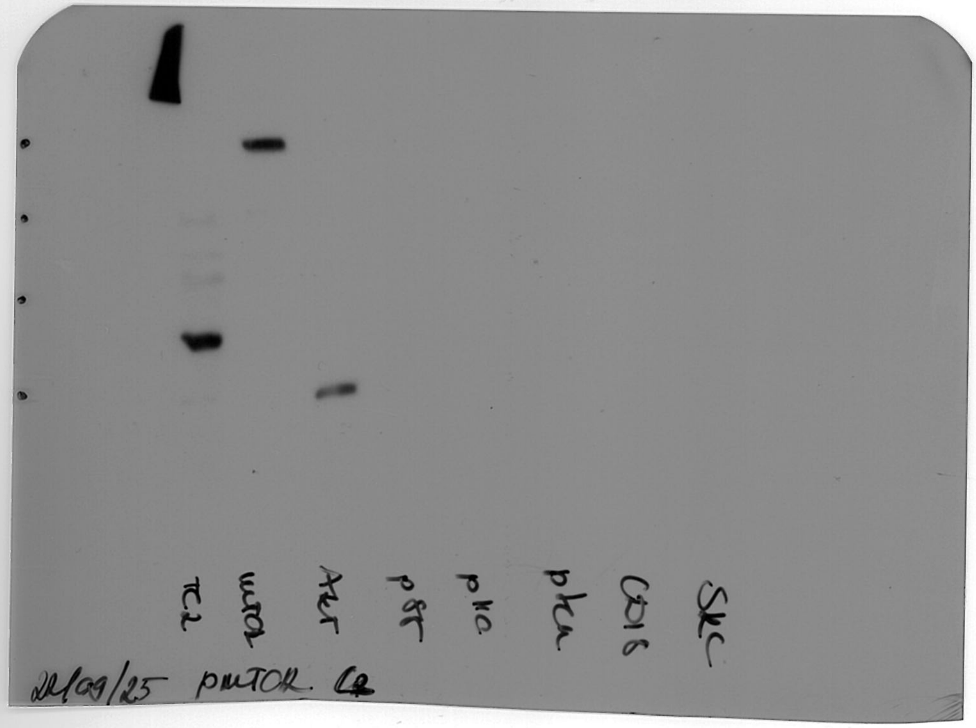
22/09/25 pmTOR. C2

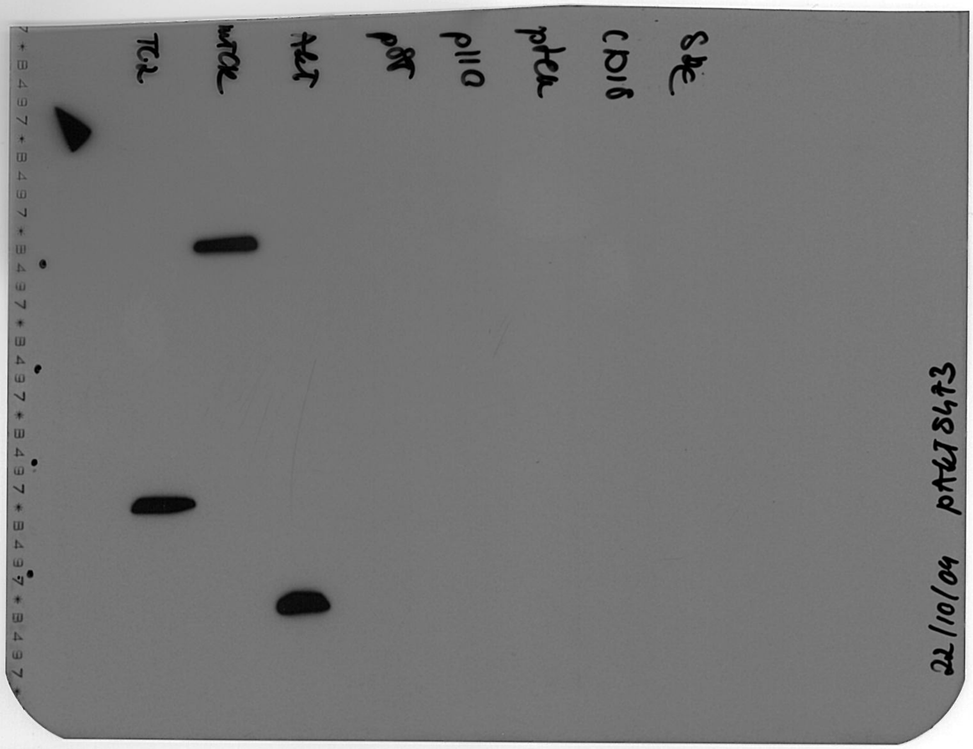
SRC
CD16
p110
p85
AKT
TCR
22/10/04 pAKT S473

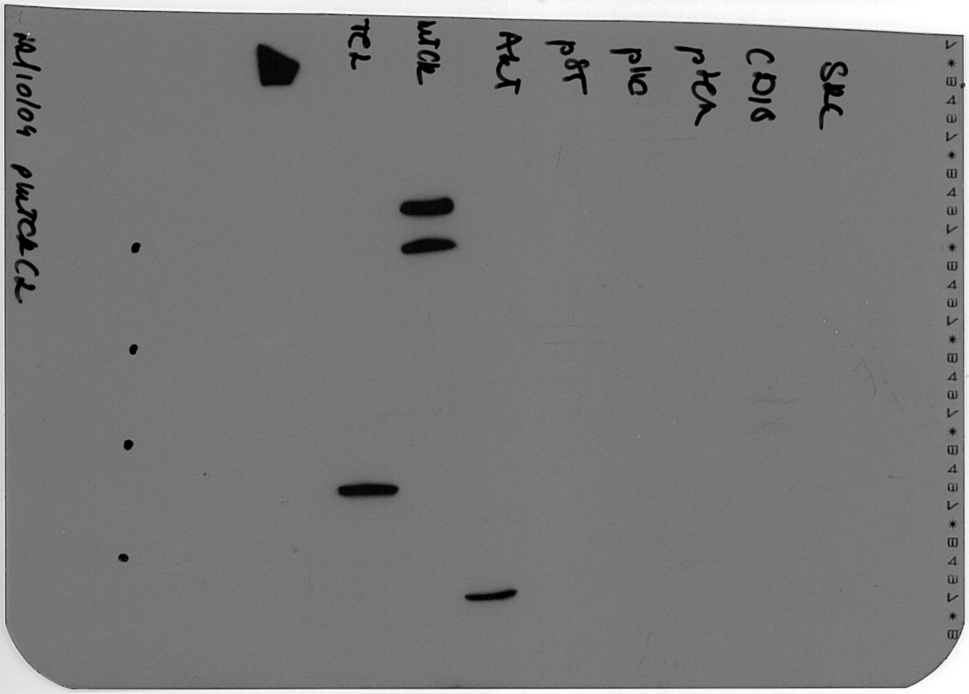
SRC
CD16
p110
p85
AKT
TCR

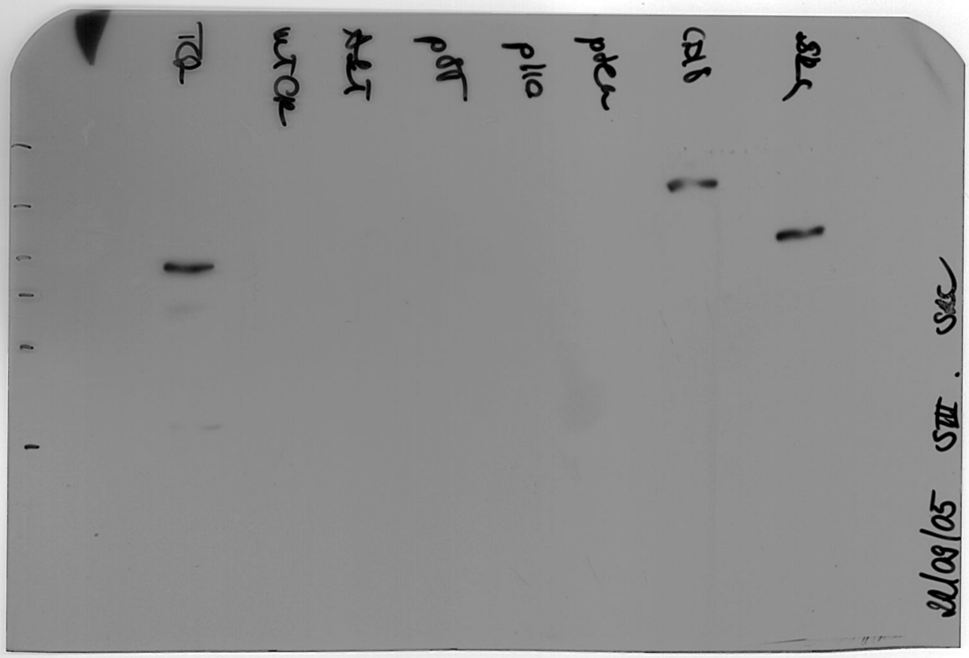

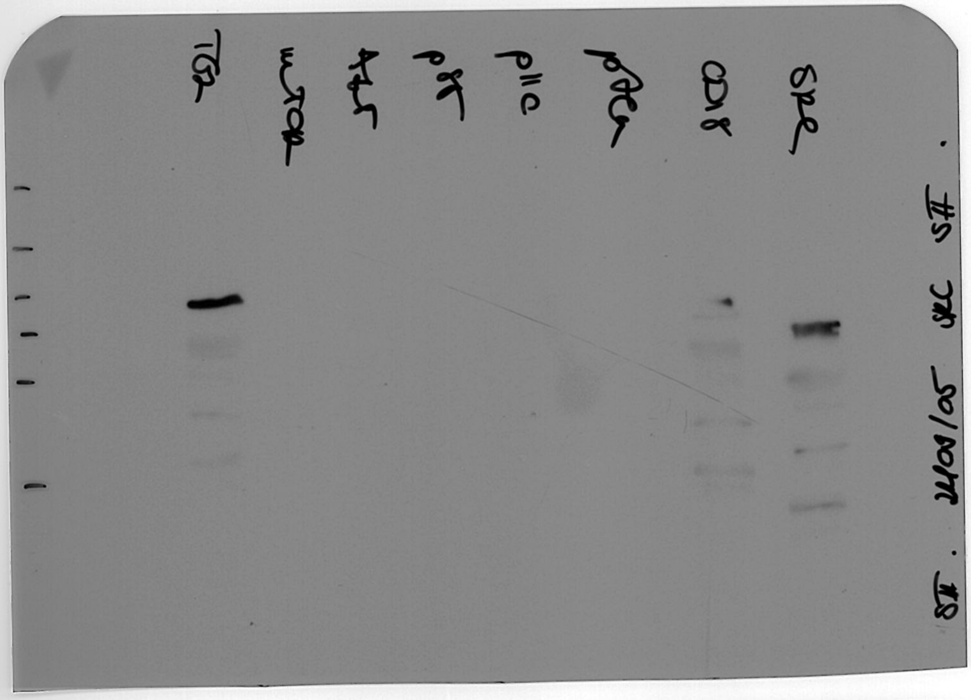

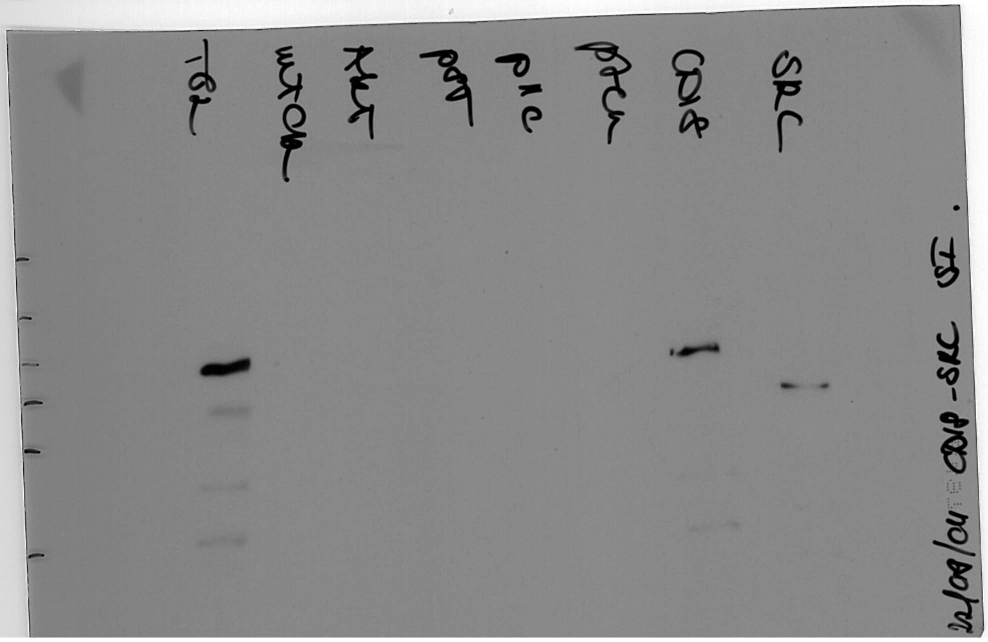

Supplement: Supplementary file 1 — Original data file [file 41419_2023_5748_MOESM1_ESM.pdf]
