# Supplementary material for: Transglutaminase 2 associated with PI3K and PTEN in a membrane-bound signalosome platform blunts cell death
Source: Cell Death Dis. 2023 Mar 28;14(3):217. doi: 10.1038/s41419-023-05748-6 (PMC10050012; doi:10.1038/s41419-023-05748-6)
Supplement: Supplementary file 2 — REVISED SUPPLEMENTARY FIGURES [file 41419_2023_5748_MOESM2_ESM.pdf]

SUPPLEMENTARY FIGURES

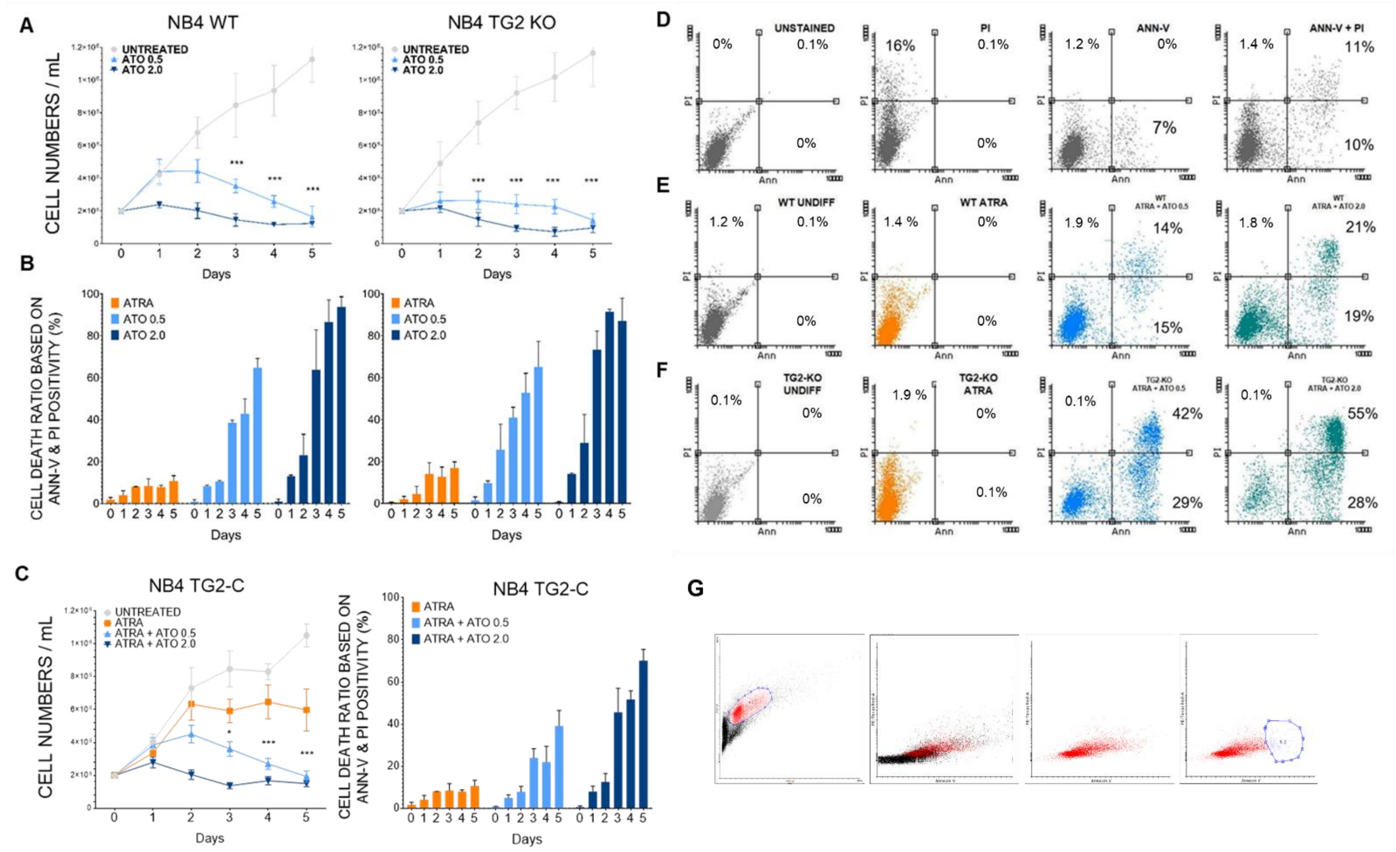

Supplementary Figure 1. ATO-induced cell death. (**A**) Cell number changes measured in KOVA Glasstic® Slide cell number counting chambers for NB4 WT, NB4 TG2-KD, and NB4 TG2-KO cells treated with 0.5 µM or 2.0 µM ATO on the indicated days (n=9). (**B**) FACS analysis of Annexin-V and PI-stained NB4 cell lines upon single ATO treatments. The percentage of cell death is represented as mean % ± SD (n=9). Measurements were conducted in triplicate; values were validated by Flowing software 2.5.1. **(C-E)** The percentage of cell death is calculated by using the quadrant method. The quadrants are representing the following populations: LL (lower left) -living cells, LR (lower right)- Annexin-V positive cells, UL (upper left)- PI positive cells, UR (upper right) – double positive cells. (**C**) The first row represents the control measurements by which the quadrant was determined. (**D-E**) Graphs represent the ATRA + ATO treated NB4 WT and NB4 TG2-KO cells raw data at day 5. Measurements were conducted in triplicates; values were validated by Flowing software 2.5.1. (**G**) Gating strategy of the Annexin-V-stained cells for cell sorting.

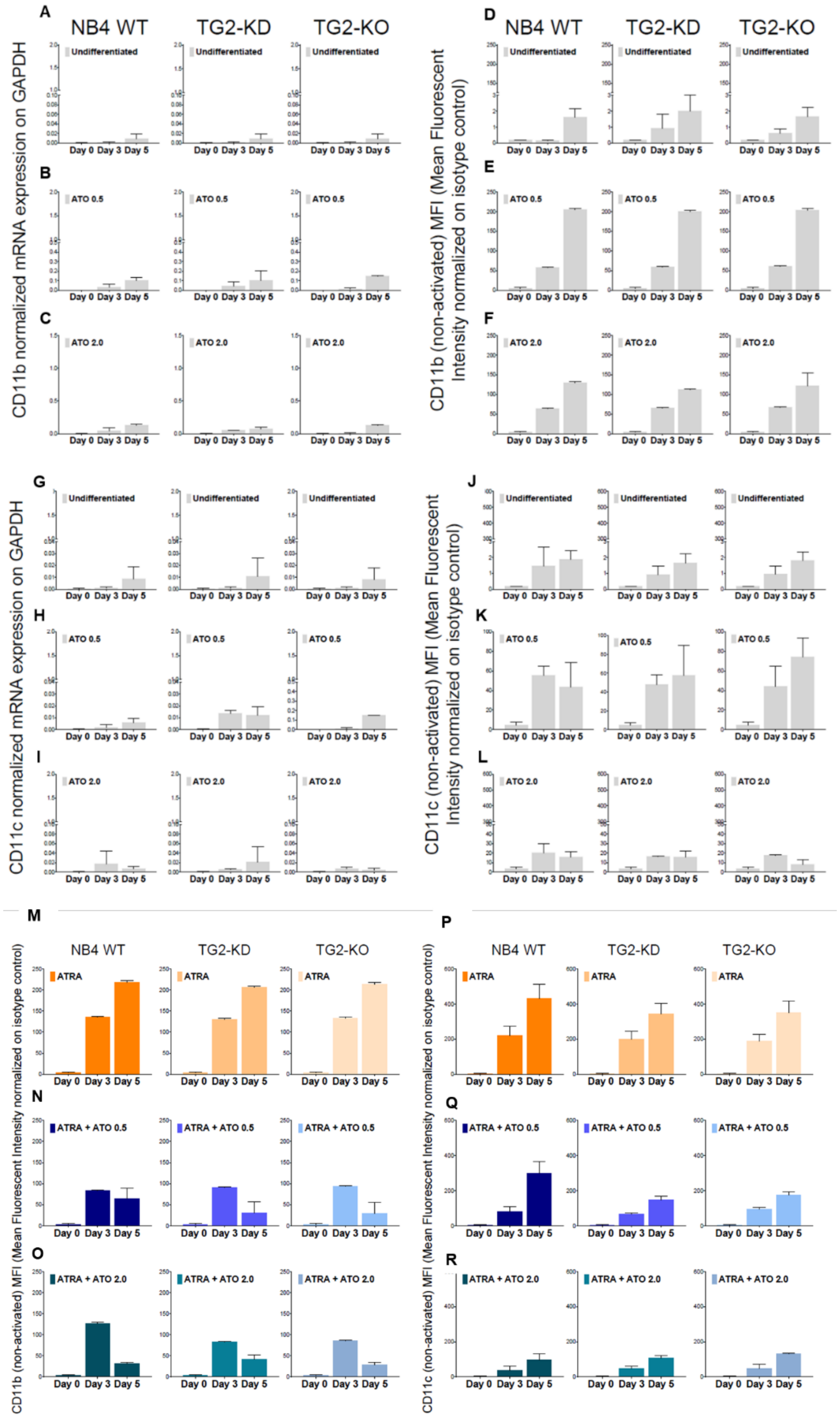

Supplementary Figure 2. Expression of "non-active" leukocyte β2 integrin CD11b/CD18 and CD11c/CD18. (**A-C**) mRNA expression of differentiation markers CD11b in untreated or ATO treated NB4 cells (n=3). (**D-F**) Flow cytometry analysis of cell surface expression of non-activated differentiation marker CD11b (n=3). (**G-I**) mRNA expression of differentiation markers CD11c in untreated or ATO treated NB4 cells (n=3). (**J-L**) Flow cytometry analysis of cell surface expression of non-activated differentiation marker CD11c/CD18 (n=3). (**M-O**) Flow cytometry analysis of cell surface expression of non-activated differentiation marker CD11b/CD18 upon ATRA + ATO treatment (n=3). (**P-R**) Flow cytometry analysis of cell surface expression of non-activated differentiation marker CD11c/CD18 upon ATRA + ATO treatment (n=3). Measurements were conducted in triplicate; values were validated by Flowing software 2.5.1.

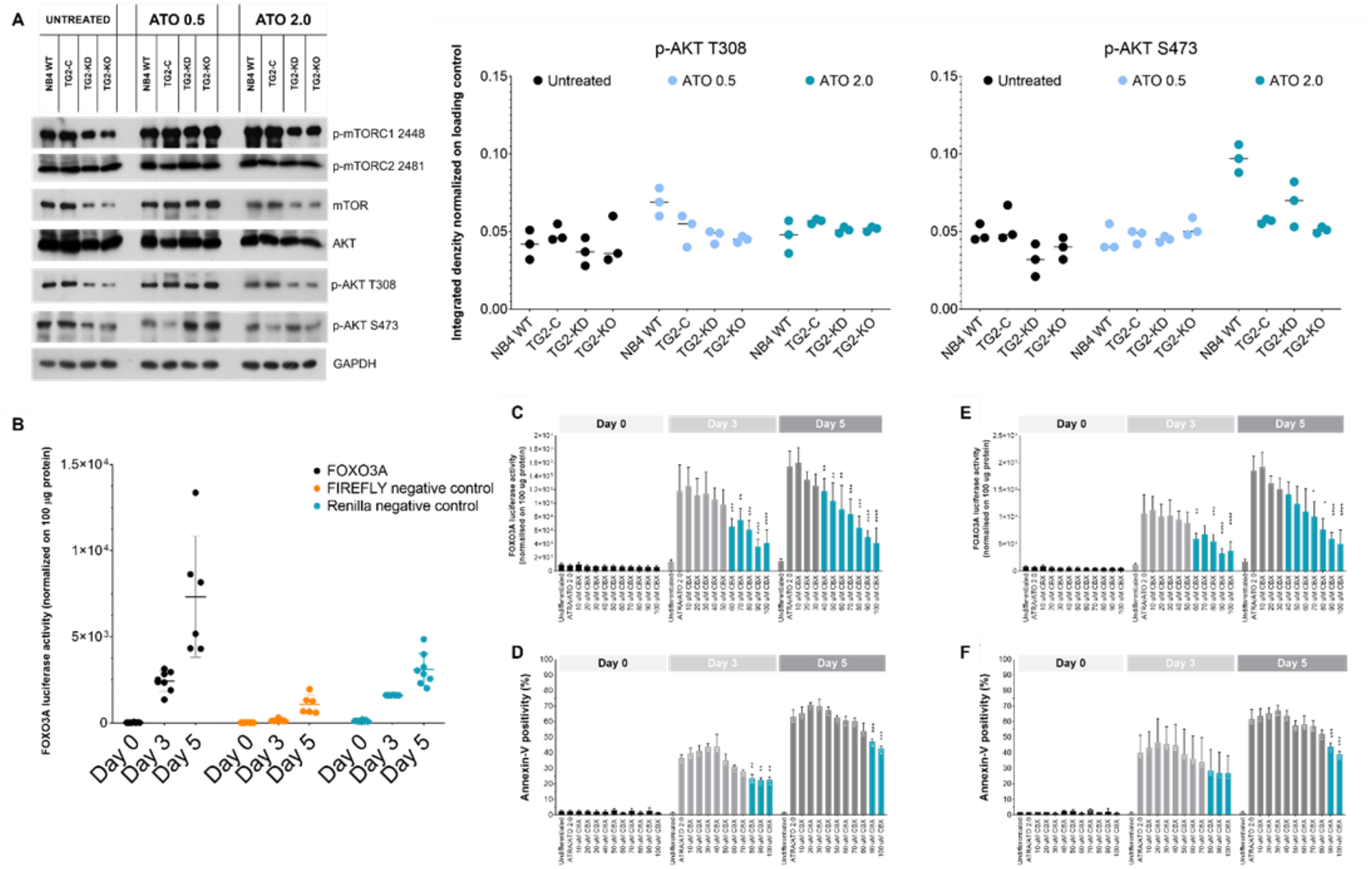

Supplementary Figure 3. CBX inhibits the FOXO3 pathway. (**A**) Representative western blot showing p-mTORC1-2 (2481, 2448) p-AKT (S473, T308), mTOR, AKT and GAPDH protein expression levels in NB4 cell lines without treatments or with ATO treatment alone (n=3). NB4 WT cells containing a FOXO3A luciferase reporter element were treated with ATRA and ATO and measured using a luminescence-based method in triplicate, with values reported as relative light units (RLU) (n=3). Values were normalized to 100 µg cell lysate protein. (**B**) FOXO3A luciferase reporter element was measured by a luminescence-based method in triplicate and reported as relative light units in NB4 WT cells using control luciferase construct: Renilla and Firefly. (RLU) (n=3). Values are normalized to 100 µg cell lysate protein. (**C-D**) NB4 WT cells (left part) and TG2-C (right part) containing a FOXO3A luciferase reporter element were treated with ATRA+ATO 2.0 with or without the FOXO3A inhibitor, CBX at different concentrations. Graph represents the percentage of cell death measured by Annexin-V and PI staining upon FOXO3A inhibition by CBX. The cyan blue color representing the statistically significant differences compared to the treatments without inhibitor. Statistical analysis was conducted by two-way ANOVA (Bonferroni post hoc test; *P<0.05, **P<0.01 and ***P<0.001, ****P<0.0001). Asterisk show the significant differences in ATRA+ATO treated vs. ATRA+ATO+CBX treated cells.

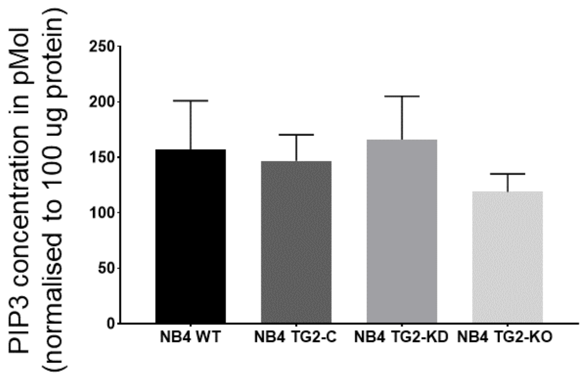

Supplement Figure 4. PIP_3 concentration in undifferentiated NB4 cells. PIP_3 levels in membranes of untreated NB4 cell lines were quantified by ELISA (Echelon Inc.). The mean values ± SD are shown for PIP_3 levels from three independent experiments. Measurements were conducted in triplicate, and the values were normalized against the protein concentration of the samples.

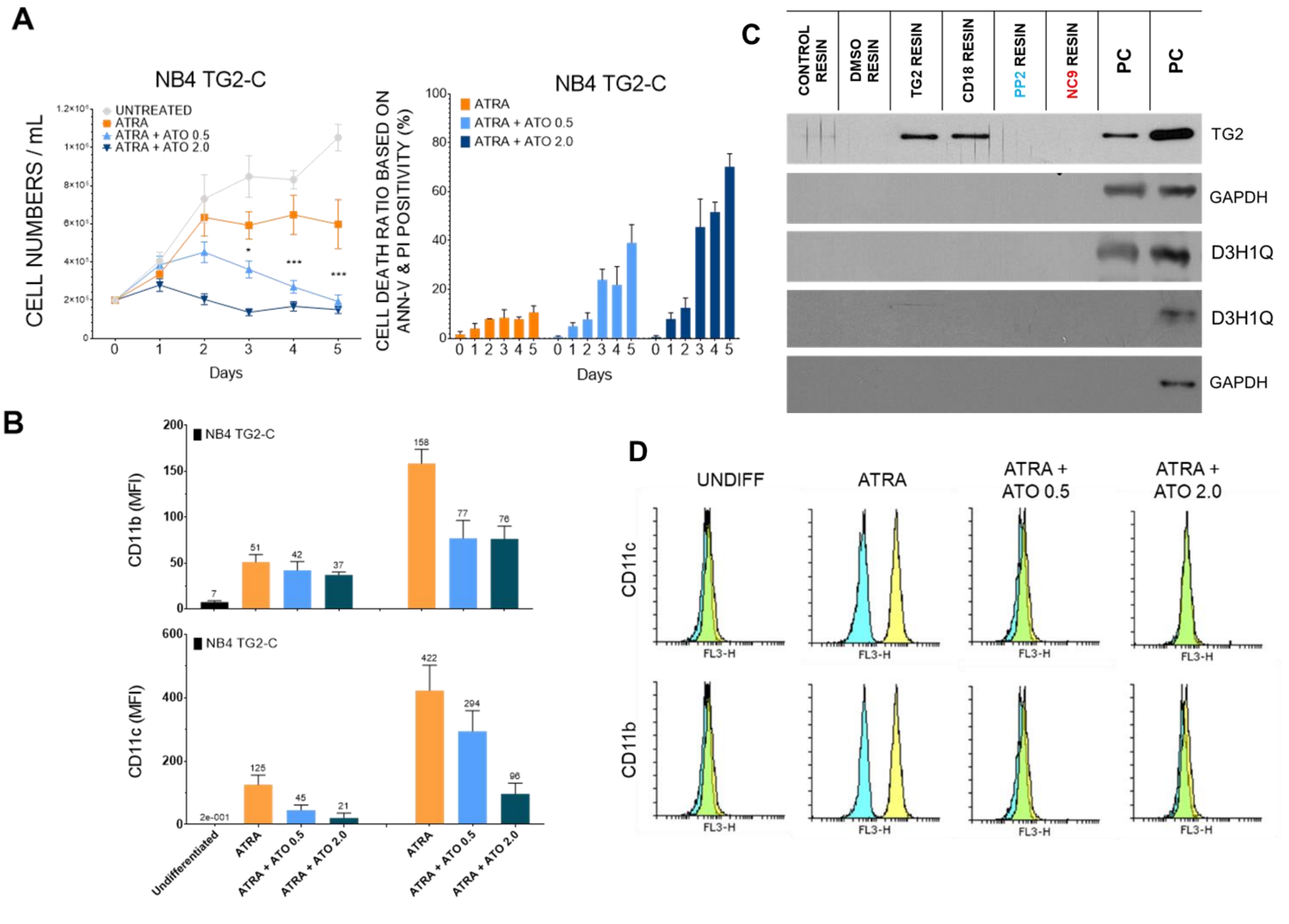

Supplementary Figure 5. ATO-induced cell death in NB4 TG2-C and control resin IP results from NB4 TG2 WT cells (A) Cell number changes measured in KOVA Glasstic® Slide cell number counting chambers for NB4 TG2-C cells without treatment or with ATRA, ATRA + ATO treatment on the indicated days (n=9). The percentage of cell death is represented as mean % ± SD (n=9). The right panel represents FACS analysis of Annexin-V and PI-stained NB4 cell lines following ATO treatments. The percentage of cell death is represented as mean % ± SD (n=9). Measurements were conducted in triplicate; values were validated by Flowing software 2.5.1.(B) Flow cytometry analysis of cell surface expression of non-activated differentiation marker CD11c/CD18 (n=3) upon ATRA + ATO treatment (n=3). Measurements were conducted in triplicate; values were validated by Flowing software 2.5.1. (C) Representative control IP-western blot showing the resin controls for DMSO, PP2, NC9 treatments. Positive control (PC) was also used as 5 days differentiated cell lysates (n=3). As a control measurements we used the expression of D3H1Q Anti-Sodium Potassium ATPase expression (D) Representative CD11c and CD11b histograms of ATRA, ATRA+ATO treated NB4 WT cells after day 5**.**

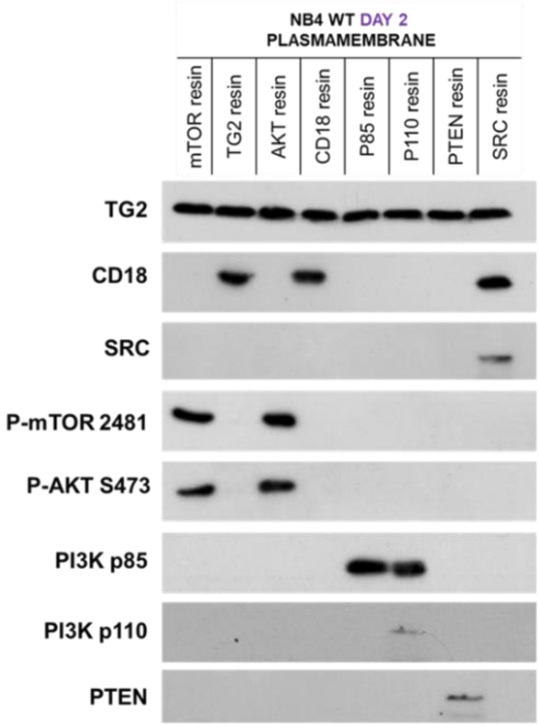

Supplementary Figure 6. Representative co-immunoprecipitation Western-blots showing plasma membrane fractions containing TG2, CD18, SRC, p-mTOR (2481), p-AKT (S473), p85, p110 and PTEN protein levels in ATRA differentiated NB4 WT cells at day 2 (n=3).
