# Supplementary material for: Transglutaminase 2 associated with PI3K and PTEN in a membrane-bound signalosome platform blunts cell death
Source: Cell Death Dis. 2023 Mar 28;14(3):217. doi: 10.1038/s41419-023-05748-6 (PMC10050012; doi:10.1038/s41419-023-05748-6)
Supplement: Supplementary file 4 — Supplementary Methods [file 41419_2023_5748_MOESM4_ESM.docx]

**SUPPLEMENTARY METHODS**

**Cell number counting**

Cell number changes in the NB4 cell lines were measured in KOVA Glasstic® Slide cell number counting chambers on the indicated days. Each number/cell/treatment/day was measured in triplicate and calculated, using the following equation: [(number cells/18grids) x 90 (factor) x 10 (dilution factor)] = cell number/mL.

**Flow cytometry analysis of apoptosis**

Approximately 4-6 × 10^5^ treated NB4 cells were harvested and washed with pre-cooled 1X PBS, followed by centrifugation at 1016 g for 3 min at 4°C. All subsequent steps were performed at 4°C Cells were washed twice with cold BioLegend Cell Staining Buffer [FITC Annexin V Apoptosis Detection Kit with PI, Biolegend], and then resuspended in Annexin-V Binding Buffer at a concentration of 0.25–1.0 × 10 cells/mL. A 100 μL volume of this cell suspension was transferred to a 5 mL test tube, and 5 μL of FITC Annexin-V was added, followed by 10 μL of propidium iodide (0.5 mg/mL). After gentle vortexing, the cells were incubated for 15 min at 4°C in the dark. In the final step, 400 μL of Annexin-V Binding Buffer was added to each tube, and the samples were analyzed by flow cytometry.

**Flow cytometric analysis of NB4 cells’ surface expression of CD11b/CD11c**

Pellets were washed and incubated with 2% BSA containing 1X PBS for 15 min and then centrifuged at 1016 *g* for 3 min. The re-pelleted material was treated with phycoerythrin (PE) and FITC or allophycocyanin (APC)-labeled CD11c/CD11b antibodies in a 1:25 dilution (R&D Systems MAB16991, Biolegend) and incubated for 2 h in the dark. Each treatment was normalized against an isotype control. After incubation with the relevant antibodies, the labeled samples were repeatedly washed and then subjected to FACS analysis (BD FACScalibur instrument, BD FACSAria™ III flow cytometer, BD Biosciences, San Jose, CA). Dead cells were excluded from the analysis by forward scatter (FSC) and side scatter (SSC) gating methods. Data were validated using Flowing software version 2.0.4, normalized, and corrected against the isotype controls for each antibody/treatment.

**Western blot analysis**

The sorted cells were pelleted and lysed in lysis buffer (50 mM TRIS, 1mM EDTA, 0.1% 2-mercaptoethanol, 0.5% Triton X-100, and 1 mM PMSF) containing a protease inhibitor cocktail (Sigma-Aldrich) with a 1:100 dilution ratio and homogenized with 5–7 strokes with a sonicator (Branson Sonifer, 450) at 40% cycle intensity. The lysed samples were centrifuged at 18213 g at 4ºC for 15 min, and the supernatant was used for protein measurements with the Bradford assay at a wavelength of 595 nm (Synergy Multi-Mode Microplate Reader). Each sample was measured in triplicate and normalized against a BSA standard (Sigma-Aldrich, 0.5 mg/mL). The protein samples were diluted to 2 mg/mL, mixed with equal volumes of 2×SDS denaturation buffer (0.125 M Tris-HCl, pH 6.8, containing 4% SDS, 20% glycerol, 10% 2-mercaptoethanol, and 0.02% bromophenol blue), and incubated at 99°C for 10 min.

Depending on their molecular weights, the proteins were separated on 8–15% SDS-polyacrylamide gels and then blotted onto a PVDF membrane (Merck-Millipore). A wet blotting method was used for mTOR proteins and a dry blotting method was used for the others. The membranes were blocked with 5% nonfat dry milk/5% BSA in Tris-buffered saline and Tween 20 (TTBS) for 1 h at RT. Primary antibodies were diluted in 0.5% milk/5% BSA in TTBS at a dilution ratio of 1:1000–1:5000 and incubated overnight at 4°C (Table 2). The membranes were washed three times with TTBS for 15 min at RT, incubated with horseradish peroxidase-labeled, affinity-purified secondary antibodies (Advansta) at 1:10000–1:20000 dilution for 1 h at RT, and then washed three times with TTBS for 15 min at RT. The targeted protein bands were visualized using an ECL Kit (Advansta). The protein bands were quantified using ImageJ software, version 1.09.

| **ANTIBODIES** | **DILUTION RATIO** |
| --- | --- |
| p-PTEN (S380/T382/383) rabbit-anti human | 1:1000 |
| PTEN rabbit-anti human | 1:5000 |
| p-mTOR (S2448) rabbit-anti-human | 1:1000 |
| p-mTOR (S2481) rabbit-anti human | 1:1000 |
| mTOR rabbit-anti human | 1:5000 |
| p-AKT (T308) | 1:5000 |
| p-AKT (S473) | 1:5000 |
| AKT | 1:5000 |
| p-PI3K p85 (Y458)/p55 (Y199) rabbit-anti human | 1:5000 |
| PI3 kinase p85 (19H8) rabbit-anti human mAb | 1:1000 |
| PI3K p110 delta subunit | 1:1000 |
| tTG anti-polyclonal | 1:5000 |
| PI3K p110beta (c-8) rabbit polyclonal | 1:5000 |
| Caspase-3 (H-277) rabbit polyclonal | 1:3000 |
| p-FOXO1/FOXO3/FOXO4 (T24/T32) | 1:5000 |
| GAPDH antimouse-human FF26A/F9 clone | 1:5000 |
| FOXO3A W15111A clone | 1:1000 |
| PTEN 4C11A11 clone | 1:5000 |

Table 2. Antibodies used for Western blotting

**Densitometry analysis**

Integrated optical densitometry was performed using ImageJ software, version 1.09. Values were normalized to the loading control of each blot/sample/lane. Blot pictures were converted to RGB 32-bit mode, and the analysis was performed using fixed rectangular selection based on gray-scale format.

**CBX treatment of NB4 WT cells**

NB4 WT cells containing a FOXO3A luciferase reporter element (QIAGEN) were treated with ATRA and ATO and Carbenoxolone (CBX) and measured in triplicate using a luminescence-based method, with values reported as relative light units (RLU). A stock solution of 1mM CBX was made and used in the FOXO3A inhibitory experiments. The CBX solution was dissolved in the RPMI 1640 cell culture media. We evaluated the FOXO3A inhibitory effect by performing a concentration dependency experiment (10-100 µM).

**PIP_3_ isolation**

The medium was aspirated from NB4 cell lines, 1 mL cold 0.5 M TCA was added, and the samples were incubated on ice for 5 min. The cells were then transferred to a 15 mL tube and centrifuged at 13,000 *g* for 5 min at 4°C. The pellets were washed with 1 mL 5% TCA/1 mM EDTA, vortexed, centrifuged again at 13,000 *g* for 5 min at 4°C, and the supernatants were discarded. The pellets were resuspended in 1 mL methanol:chloroform (2:1), vortexed 3–4 times over 10 min at RT, centrifuged again at 13,000 *g* for 5 min at 4°C, and the pellets were suspended again in 0.5 mL chloroform:methanol:12 N HCl (40:80:1). The samples were incubated for 15 min with occasional vortexing, and then 180 μL chloroform was added, followed by 320 μL 0.1 N HCl. The samples were centrifuged at 13,000 *g* for 5 min at 4°C, and the organic (lower) phase was transferred to a new 1.5 mL tube, followed by the addition of 30 μL 0.1 M ammonium hydroxide in methanol. The neutralized organic phase was then dried in a vacuum dryer.

**Annexin-V labeling of NB4 cells and live/dead cell sorting**

Approximately 1–2 × 10^6^ NB4 treated cells were harvested and washed with pre-cooled 1X PBS, followed by centrifugation at 55 *g* for 3 min at 4°C. All subsequent steps were performed at 4°C. The cells were labeled with FITC-conjugated Annexin-V (Biolegend) for 15 min in the dark. Excess dye was removed by centrifugation at 55 *g* for 3 min, and the pelleted cells were resuspended in pre-cooled PBS. The cells were analyzed and sorted using a BD FACSAria™ III flow cytometer (BD Biosciences, San Jose, CA). The sorted cells were used for western blot analysis.

The apoptotic features were evaluated by first sorting for the size and granularity features of the NB4 cells, followed by gated cell population filtering against the FITC-positive cells. The FITC-positive cells were sorted into a separate 15 mL tube. For each cell line, a minimum of 1 × 10^5^ cells were sorted based on their FITC positivity.

**Gene expression**

The methods for the isolation of RNA and RT-PCR/RT-QPCR have been published previously**^17,18^**. The real-time Q-PCR reaction was performed using the following TaqMan probes (ABI, Applied Biosystems): TG2, PTEN, PI3K-p85, PI3K-p110, human ALU, and GAPDH (Thermo Fisher Scientific) (Table 1). The analysis was carried out using a Roche LightCycler® 480 II (Roche Molecular Systems, Inc.)

| **OLIGO** | **Assay ID** |
| --- | --- |
| TGM2 | Hs01096680_m1 |
| PTEN | Hs02621230_s1 |
| PI3K-p85 | Hs00388782_m1 |
| PI3K-p110 | Hs00898499_m1 |
| Human-ALU sequence | Forward primer TGGTGGCTCTCTCCTGTAAT |
| Human-ALU probe | TGAGGCAGGAGAATCGCTTGAACC FAM-MGB |
| Human-ALU sequence | Reverse primer GATCTCGGCTCACTGCAAC |
| GAPDH | Mm01328877_g1 |
| CYCLOPHILIN-D | Mm01328877_g1 |
| ACTIN | Hs01101944_s1 |

*Table 1. Oligos used in RT-QPCR*

**Plasma membrane preparation**

The plasma membrane was isolated according to the Abcam protocol (ab65400). The cells were harvested, centrifuged (1016 g for 5 min), washed once with 3 mL of ice-cold PBS, and resuspended in 2 mL of the Homogenization Buffer Mix in an ice-cold Dounce homogenizer. The cells were homogenized on ice 70–75 times, and the homogenate was transferred to 1.5 mL microcentrifuge tubes. The tubes were centrifuged at 700 *g* for 10 min at 4°C, and the supernatants were transferred to new vials for centrifugation at 10,000 *g* for 30 min at 4°C. The resulting supernatant was the cytosol fraction, while the pellet was the total cellular membrane protein fraction (containing proteins from both the plasma membrane and cellular organelle membranes). The total membrane protein pellet was resuspended in 200 μL of the Upper Phase Solution, 200 μL of the Lower Phase Solution was added, and the solution was mixed well and incubated on ice for 5 min. Another 200 μL of Upper Phase Solution and 200 μL of Lower Phase Solution were added, followed by centrifugation at 1000 *g* for 5 min at 4 ºC. The upper phase was collected, diluted in 5 volumes of water, kept on ice for 5 min, and 18213 g in a microcentrifuge tube for 10 min at 4°C. The resulting pellet was the plasma membrane protein fraction.

***In vivo* mouse experiment**

All experiments were performed according to the guidelines of the Institute for Laboratory Animal Research, University of Debrecen, Faculty of Medicine, and were approved by the national and institutional ethics committee for laboratory animals used in experimental research (Project ID:4/2020/DEMAB).

NB4 WT and NB4 TG2-KO cells (1×10^7^) were washed with sterile PBS and injected into the retro-orbital region of 8–9-week-old NOD/SCID (CB17/lcr-Prkdc^scid^, JANVIER LABS) female mice under sterile conditions.

Experiments were carried out with four groups of treatments:

1. Control (1:1 DMSO:Ethanol [v/v])

2. All-trans retinoic acid (ATRA) at a 1 µM final concentration + Dexamethasone (DEX; 50 μg) (the ATRA powder stock solution was prepared in DMSO: Ethanol)

3. Arsenic trioxide (ATO) at a 2.0 µM final concentration

4. ATRA 1 µM + ATO 2.0 µM

Anesthetized mice were administered 1.0 mg/kg ATRA + 50 μg DEX, 0.75 mg/kg ATO, or a combination of ATRA + ATO intraperitoneally every 2 days for 14 days. Blood samples were harvested from the tail veins at the beginning of treatment and again at the termination on day 14. A 27.5-gauge or 0.5-gauge insulin needle/syringe (Terumo U-100, Terumo Medical Corporation, Elkton, MD) was used for the i.p. injections of the treatments. As an experimental control, the DMSO:Ethanol vehicle used to prepare the ATRA powder stock solution was administered i.p. The DMSO amount (v/v) was kept below 2% to avoid any cytotoxic effects. At the end of treatment at day 14, the mice were anesthetized with isoflurane. Circulating human NB4 and mouse cells were analyzed from total cardiac blood samples by flow cytometry and distinguished by human and mouse neutrophil surface-expressed marker proteins.

**Human CD11c/CD11b/Annexin-V positive labeling of mouse blood cells**

Blood was collected from mouse hearts at day 14 and treated with sodium citrate (8% v/v) to prevent coagulation. Blood samples were lysed with lysis buffer (BD PHarmn LyseTM) following the kit manufacturer’s protocol. The lysed samples were centrifuged at 1016 *g* for 3 min and then incubated for 2 h in the dark with the PE and FITC or APS-labeled CD11c/CD11b antibody at a 1:25 dilution ratio (R&D Systems). The samples were labeled with F4/80 antibody (Biolegend) in a 1:100 dilution ratio for 15 min in the dark at 4°C to detect mouse macrophages. F4/80 positive mouse cells were excluded, and the human NB4 cell population was gated out. CD11 positive cells were then gated out from the total human cell population, and APC labeling was used to filter out the Annexin-V positive cells, resulting in a CD11 and Annexin-V positive cell population. The cells were then centrifuged at 1016 *g,* and the supernatant was discarded. The cells were resuspended in Annexin-V binding buffer, labeled with APC conjugated Annexin-V (Biolegend) for 15 min at 4°C in the dark (1:100 dilution), and then subjected to flow cytometry (BD FACSAria™ III).

The cells were first gated based on their size and granularity (FSC and SSC). From the total cell population, F4/80 positive mouse cells were excluded, and the human NB4 cell population was gated out. The CD11-positive cells were then gated out from the total human cell population, and the Annexin-V positive cells were filtered out based on their APC labeling, resulting in a CD11-positive, Annexin-V positive cell population. The FACS data were validated by Flowing software 2.0.5 version and normalized and corrected to their respective isotype controls.

**Procedure for co-Immunoprecipitation**

**Antibody immobilization**

AminoLink Plus Coupling Resin Dynabead (Thermo Scientific, Waltham, MA, USA) and all the reagents were equilibrated to room temperature before the immunoprecipitations. Each co-IP reaction used 2 mL of 1X Coupling Buffer, prepared by diluting the 20X Coupling Buffer with ultrapure water. The AminoLink Plus Coupling Resin was suspended using a wide-bore or cut pipette tip, and 50 μL of the resin slurry was transferred into a Pierce Spin Column. The column was placed into a microcentrifuge tube, centrifuged at 1000 *g* for 1 min, and the flow-through was discarded. The resin was washed 2 times with 200 μL of 1X Coupling Buffer, centrifuged, and the flow-through discarded. Coupling was performed by adding 10–75 μg of affinity-purified antibody in 200 μL 1X Coupling Buffer. The amounts of each antibody used are listed in Table 3.

| **Type of ANTIBODY** | **Amount used (μg)** |
| --- | --- |
| TG2 | 50 |
| SRC | 25 |
| CD18 | 30 |
| AKT | 50 |
| mTOR | 75 |
| PTEN | 25 |
| P85 | 50 |
| P110 | 50 |

*Table 3. Antibody amounts used for immobilization*

In a fume hood, 3 μL of the supplied Sodium Cyanoborohydride Solution was added for every 200 μL of reaction volume. The samples were incubated on a rotator or mixer at room temperature for 90–120 min, ensuring that the slurry remained suspended during incubation. The antibody solution was removed, and the resin was washed twice with 1X Coupling Buffer, followed by the addition of 200 μL of Quenching Buffer. The column was centrifuged, the flow-through was discarded, and another 3 μL of Sodium Cyanoborohydride Solution was added in the fume hood and incubated for 15 min with gentle shaking. The resin containing the bound antibody was washed twice with 200 μL of 1X Coupling Buffer, centrifuging after each wash. The resin was then washed six times with 150 μL of Wash Solution. For every 1 mg of cell lysate, 80 μL of the Control Agarose Resin slurry was added (40 μL of settled resin) to a spin column, the column was centrifuged to remove the storage buffer, 100 μL of 1X Coupling Buffer was added, and the column was centrifuged. Lysate (1 mg) was added to the resin column and incubated overnight at 4ºC with gentle mixing. The column was then centrifuged at 1000 *g* for 1 min, and the resin was discarded. The flow-through was saved and added to the immobilized antibody for the co-IP.

**Co-IP**

All Co-IP steps were performed at 4°C. The protocol used the IP Lysis/Wash Buffer for coupling and washing the immune complex. The supplied 20X Modified Dulbecco’s PBS was used as an alternative binding/wash buffer. For each co-IP reaction, 2 mL of 1X Modified Dulbecco’s PBS was prepared by diluting it with ultrapure water. The resin was washed twice by adding 200 μL of IP Lysis/Wash Buffer to the spin column containing the antibody-coupled resin and centrifuging. The lysate mixture and controls were added to the appropriate resins and incubated overnight at 4ºC with gentle shaking. Each sample was centrifuged, and the flow-through was collected. Each sample was washed two more times with 200 μL IP Lysis/Wash Buffer and centrifuged after each wash. Elution Buffer (10 μL) was added and centrifuged, followed by addition of 50 μL of Elution Buffer and incubation for 15 min at RT. The flow-through was collected and analyzed by LC-MS/MS.

**In-gel digestion of proteins**

The protein bands were excised from the gel and subjected to in-gel trypsin digestion. The bands were destained using a 1:1 ratio of 25 mM ammonium bicarbonate (pH 8.5) and 50% acetonitrile, followed by the reduction of the proteins using 20 mM dithiothreitol (Sigma, St. Louis, MO, USA) for 1 h at 56°C. The samples were then alkylated with 55 mM iodoacetamide (Sigma, St. Louis, MO, USA) for 45 min in the dark. Overnight trypsin digestion was carried out with 100 ng stabilized MS-grade trypsin (ABSciex, Framingham, MA, USA) at 37°C. The reaction was stopped by the addition of concentrated formic acid. The tryptic peptides were extracted from the gel pieces, dried in a vacuum concentrator (Thermo Scientific, Waltham, MA, USA), and kept at -20°C until mass spectrometry analysis.

**Liquid chromatography-mass spectrometry analysis**

Proteins were identified by liquid chromatography with tandem mass spectrometry by dissolving the peptides in 10 μL 1% formic acid (VWR Ltd., Radnor, PA, USA) and separating them using a 180 min water/acetonitrile gradient on an Easy nLC 1200 nano UPLC (Thermo Scientific, Waltham, MA, USA). The peptide mixtures were desalted in an Acquity UPLC Symmetry C18 trap column (20 mm × 180 µm, 5 μm particle size, 100 Å pore size; Waters, Milford, MA, USA), followed by separation in a nanoAcquity Peptide BEH C18 analytical column (150 mm × 75 μm, 1.7 μm particle size, 130 Å pore size; Waters, Milford, MA, USA). The chromatographic separation was performed using a gradient of 5–7% solvent B over 5 min, followed by 15% solvent B over 50 min, then 35% solvent B over 60 min, then increasing solvent B to 40% over 28 min, then to 85% over 5 min, followed by 85% over 10 min, and then a return to 5% solvent B in 1 min and a 16 min hold. Solvent A was 0.1% formic acid in LC water (Sigma, St. Louis, MO, USA); solvent B was 95% acetonitrile (Sigma, St. Louis, MO, USA) containing 0.1% formic acid. The flow rate was set at 300 nL/min.

Data-dependent acquisition experiments were carried out on an Orbitrap Fusion mass spectrometer (Thermo Scientific, Waltham, MA, USA). The 14 most abundant multiply charged positive ions were selected from each survey MS scan using a scan range of 350–1600 m/z for MS/MS analyses (Orbitrap analyzer resolution: 60.000, AGC target: 4.0e5, acquired in profile mode). Collision-induced dissociation (CID) fragmentation was performed in the linear ion trap mode with 35% normalized collision energy (AGC target: 2.0e3, acquired in centroid mode). Dynamic exclusion was enabled during the cycles (exclusion time: 45 s).

**Protein identification**

The acquired LC-MS/MS data were used for protein identification using MaxQuant 2.0.1 software] and searching against the Human SwissProt database (release: 2021.04, 20376 sequence entries) and against the contaminants database provided by the MaxQuant software. Cys carbamidomethylation, Met oxidation, and N-terminal acetylation were set as variable modifications. A maximum of two missed cleavage sites were allowed. The results were imported into Scaffold 5.0.1 software (Proteome Software Inc., Portland, OR, USA). Proteins were accepted with at least 2 identified peptides using a 1% protein false discovery rate (FDR) and 95% peptide probability thresholds.

**Sample preparation for western blotting**

5X Lane Marker Sample Buffer was equilibrated to room temperature. The samples were gently mixed with the sample buffer by inverting 5–10 times. The samples were heated at 95–100°C for ~5 min.

**Far-western blotting**

Cell membrane lysates of differentiated NB4 cells were used for far-western blotting. We generated 8 different resins with the following antibodies: TG2, mTOR, AKT, p85, p110, PTEN, CD18, and SRC. Immunoprecipitation was conducted as described above in the section “Immunoprecipitation.”

The 8 different eluates containing the fished-out proteins (TG2, mTOR, AKT, p85, p110, PTEN, CD18, and SRC) were subjected to electrophoresis on 10% SDS gel our samples with constant 120 V for 2 h, allowing the lower part of the marker to run out. The gel was electroblotted with a semi-dry method onto a PVDF membrane at 25 V for 30 min. The PVDF membrane was dried and stained with Ponceau S for 10 min at room temperature. After washing with MilliQ water, the proteins were renatured using Guanidine-HCl overnight (Table 4).

*Table 4. AC buffer (Acetyl buffer) containing guanidine–HCl for renaturing.*

Concentration of guanidine–HCl (M) 0.1

Glycerol (mL) 2.5

5 M NaCl (mL) 0.5

1 M Tris, pH 7.5 (mL) 0.5

0.5 M EDTA (mL) 0.05

10% Tween-20 (mL) 0.25

Guanidine–HCl (8 M) (mL) 0.31

Milk powder (g) 0.5

1 M DTT (µL) 25

ddH_2_O (mL) 20.89

Total volume (mL) 25

After renaturation, the membrane was blocked with 5% milk in PBST buffer for 1 h at RT.

**Incubation of the PVDF membrane with interacting prey protein(s)**

The PVDF membrane containing the isolated plasma membrane fractions was incubated overnight at 4ºC with 5 mL of diluted plasma membrane lysate (total protein content was 40 µg in 5 mL). After the incubation, we used 25 mM disuccinimidyl suberate (DSS), a crosslinking agent, for 30 min at room temperature to fix the protein–protein interactions on the membrane.

**Detection of prey proteins bound to bait proteins on the blot**

After three washes with PBST buffer, each for 10 min, to remove the unbound prey protein(s), we incubated the blot with an appropriate diluted primary antibody for 1 h at RT in 5% milk in the PBST buffer. Incubation with the primary antibody was carried out overnight at 4 ºC.

After 3 washes with PBST buffer, each for 10 min, the membrane was treated with secondary antibody (HRP-conjugated anti-mouse/rabbit/rat IgG antibody; 1:10,000 dilution) for 1 h at RT. After another 3 washes with the PBST, each for 10 min, the membrane was rinsed with PBS for 5 min. Chemiluminescent detection of the bound prey protein was performed using an ECL kit (Advansta), according to the manufacturer’s instructions.

**Incubation of the membrane with purified interacting (bait) protein(s)**

The membrane containing the isolated membrane fractions was incubated overnight at 4ºC. In our experiments, we applied 5 mL of diluted membrane fraction (total protein content was 40 µg in 5 mL). After the incubation, we used 25 mM disuccinimidyl suberate (DSS), a crosslinking agent, for 30 min at room temperature to fix the protein–protein interactions on the membrane.

**Detection of pray proteins bound to bait proteins on the blot**

After three washes with PBST buffer, each for 10 min, to remove the unbound pray protein(s), we incubated the blot with an appropriate diluted primary antibody for 1 h at RT in 5% milk in the PBST buffer. Incubation with the primary antibody was carried out overnight at 4 ºC.

After 3 washes with PBST buffer, each for 10 min, the membrane was treated with secondary antibody (HRP-conjugated anti-mouse/rabbit/rat IgG antibody; 1:10,000 dilution) for 1 h at RT. After another 3 washes with the PBST, each for 10 min, the membrane was rinsed with PBS for 5 min. Chemiluminescent detection of the bound pray protein was performed using an ECL kit (Advansta), according to the manufacturer’s instructions.
